# MAAPER: model-based analysis of alternative polyadenylation using 3′ end-linked reads

**DOI:** 10.1186/s13059-021-02429-5

**Published:** 2021-08-10

**Authors:** Wei Vivian Li, Dinghai Zheng, Ruijia Wang, Bin Tian

**Affiliations:** 1 Department of Biostatistics and Epidemiology, Rutgers School of Public Health, Rutgers, The State University of New Jersey, Piscataway, NJ 08854 USA; 2 Department of Microbiology, Biochemistry and Molecular Genetics, Rutgers New Jersey Medical School, Newark, NJ 07103 USA; 3 Program in Gene Expression and Regulation, and Center for Systems and Computational Biology, The Wistar Institute, Philadelphia, PA 19104 USA

**Keywords:** Alternative polyadenylation, RNA sequencing, Bioinformatic tool, 3′ end reads, Cellular stress, Trophoblasts

## Abstract

Most eukaryotic genes express alternative polyadenylation (APA) isoforms. A growing number of RNA sequencing methods, especially those used for single-cell transcriptome analysis, generate reads close to the polyadenylation site (PAS), termed *nearSite* reads, hence inherently containing information about APA isoform abundance. Here, we present a probabilistic model-based method named MAAPER to utilize nearSite reads for APA analysis. MAAPER predicts PASs with high accuracy and sensitivity and examines different types of APA events with robust statistics. We show MAAPER’s performance with both bulk and single-cell data and its applicability in unpaired or paired experimental designs.

**Supplementary Information:**

The online version contains supplementary material available at 10.1186/s13059-021-02429-5.

## Introduction

Well over half of the eukaryotic protein-coding genes and many long non-coding genes harbor multiple cleavage and polyadenylation sites, or PASs, resulting in expression of alternative polyadenylation (APA) isoforms [1, 2]. While most of the APA events occur in the 3′-most exon of genes, leading to isoforms with variable 3′ untranslated regions (3′UTRs), a sizable fraction of APA sites, e.g., ~20% in the human genome, are located in regions upstream of the last exon, almost exclusively in introns. Intronic polyadenylation (IPA) leads to alternation of coding sequence (CDS) as well as 3′UTR. APA is increasingly being appreciated as a major mechanism for gene regulation [1, 2], diversifying the transcriptome in different cell types and under various pathological and physiological conditions. Accurate and sensitive profiling of APA isoforms is of great importance in understanding the mechanisms and consequences of APA and can have diagnostic values in clinical settings [3].

The advent of RNA sequencing (RNA-seq) technologies has enabled comprehensive transcriptome analysis. RNA-seq data have also been used to examine APA isoform profiles, either by taking advantage of RNA-seq read coverage changes at the PASs [4] or by using annotated PASs [5–7]. However, because regular RNA-seq data are not designed to identify PASs, these approaches lack high accuracy and sensitivity for PAS identification and APA profiling.

**Fig. 1:**
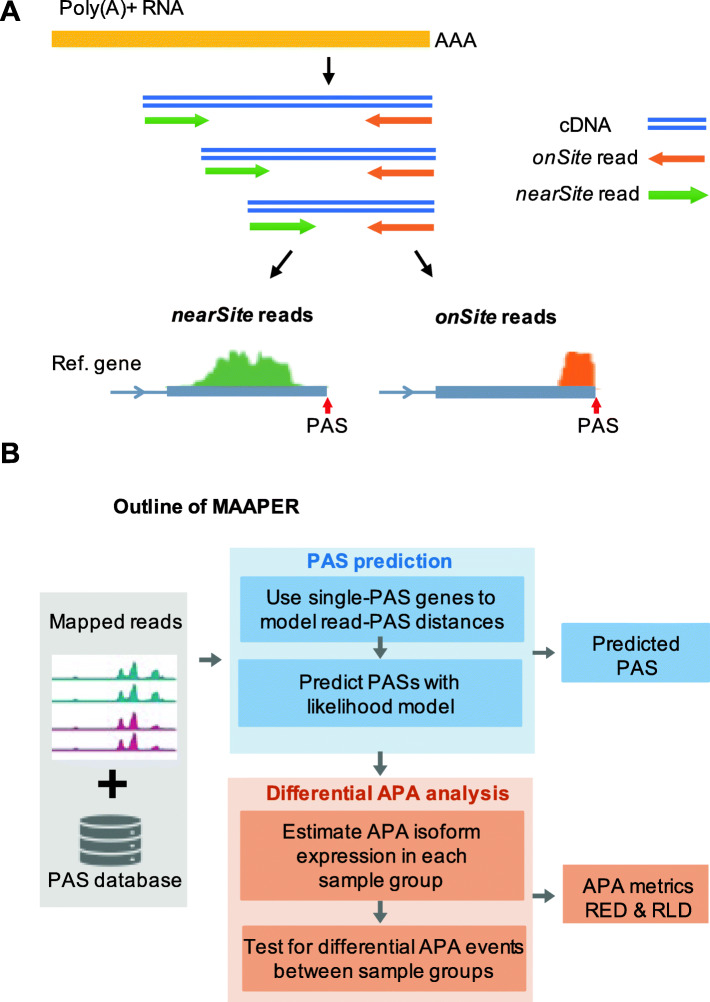
Overview of the MAAPER method. **A** A schematic illustrating the generation of *nearSite* reads (e.g., QuantSeq FWD, 10x Genomics) and *onSite* reads (e.g., QuantSeq REV). **B** Outline of the MAAPER method. MAAPER takes aligned *nearSite* reads and a PAS database (polyA_DB) and outputs predicted PAS positions and APA analysis results. Two APA metrics are generated, namely, relative expression difference (RED) and relative lengthen difference (RLD)

RNA sequencing methods that generate reads biased to the 3′ end of transcripts, collectively called 3′ end sequencing, are increasingly being used to study gene expression in bulk RNA samples or in single cells [8]. Some methods generate reads that end at the PASs, such as PAS-seq [9], PolyA-seq [10], 3′-seq [11], and QuantSeq REV [12]. For simplicity, we name those reads *onSite* reads. As such, the PASs can be directly identified after mapping the reads to the reference genome (Fig. 1A). While straightforward for PAS identification, these methods typically require a custom primer containing an oligo(T) region for sequencing [12], limiting their applications. By contrast, some other methods generate reads that are close to but do not necessarily contain the PASs, such as QuantSeq FWD [12] and PAT-seq [13] (Fig. 1A). For simplicity, those reads are named *nearSite* reads. Conceivably, nearSite reads are “3′ end-linked”, because they contain information about the PAS positions. Notably, several 3′ end counting protocols used in single-cell RNA sequencing (scRNA-seq), such as 10x Genomics [14] and Drop-Seq [15], also generate nearSite reads [16, 17]. As such, the number of nearSite read data sets has increased drastically in recent years because of the rapid adoption of scRNA-seq methods. However, mining nearSite reads for APA analysis has hitherto been limited due to lack of suitable methods. The major challenge of using nearSite reads for APA analysis is accurate assignment of reads to specific PASs. Here we present a computational method named MAAPER (model-based analysis of alternative polyadenylation using 3′ end-linked reads), which accurately assigns nearSite reads to PASs through statistical modeling and generates multiple statistics for APA analysis.

## Results

### Design of MAAPER

While nearSite reads do not directly map to PASs, they inherently contain information about the PASs that give rise to the reads. We reasoned that the distance between the 5′ end of a nearSite read and PAS reflects the cDNA fragment size of a sequencing library and thus could be statistically modeled. We further reasoned that genes containing only a single PAS would provide a clear picture for studying the read-PAS relationship, and the derived information can be useful to examine genes containing multiple PASs. To these ends, we developed a strategy to first predict PASs (stage 1) and then use them for APA analysis (stage 2). We named our method MAAPER (Fig. 1B).

At the PAS prediction stage, MAAPER first uses genes that contain only a single PAS to learn the distribution of read-PAS distance (Fig. 1B). PolyA_DB database, a comprehensive PAS database we previously constructed [18], is used to identify those genes (Additional file 1: Figure S1). It is worth noting that the current version (v3) of PolyA_DB contains PASs identified by the highly accurate 3′ end sequencing method, 3′ end extraction and sequencing (3′READS) [19], ensuring data quality. Statistical learning is carried out with a Gaussian kernel function for non-parametric estimation of read-PAS distances, which captures the distributional characteristic of the distances without assuming data uniformness. Next, based on the learned read-PAS relationship, a likelihood model predicts PASs for each nearSite read mapped to a given gene. As such, each read has a set of probability scores indicating the likelihood for all PASs of the gene annotated in PolyA_DB (Additional file 1: Figure S2).

At the APA analysis stage, MAAPER uses the predicted PASs for quantitative analysis of APA difference between samples (Fig. 1B). For each gene, the expression of a given PAS is based on the proportion of corresponding RNA isoform in a sample. Two types of statistics are generated, relative expression difference (RED) and relative length difference (RLD). With RED, the relative abundance of two APA isoforms with the greatest change in expression levels are compared across samples. With RLD, the relative lengths of all APA isoforms weighted by expression levels are compared across samples. These two scores are generated for 3′UTR APA and intronic APA, separately (see below and the “Materials and methods” section).

To generate *P* values, MAAPER uses a likelihood ratio test (LRT) to determine if a gene’s overall PAS profile differs between samples and a Fisher’s exact test is used to determine if two selected PAS differ in their isoform abundance between samples. Both paired and unpaired tests are available (see below and the “Materials and methods” section).

### Accurate and sensitive PAS prediction by MAAPER

**Fig. 2:**
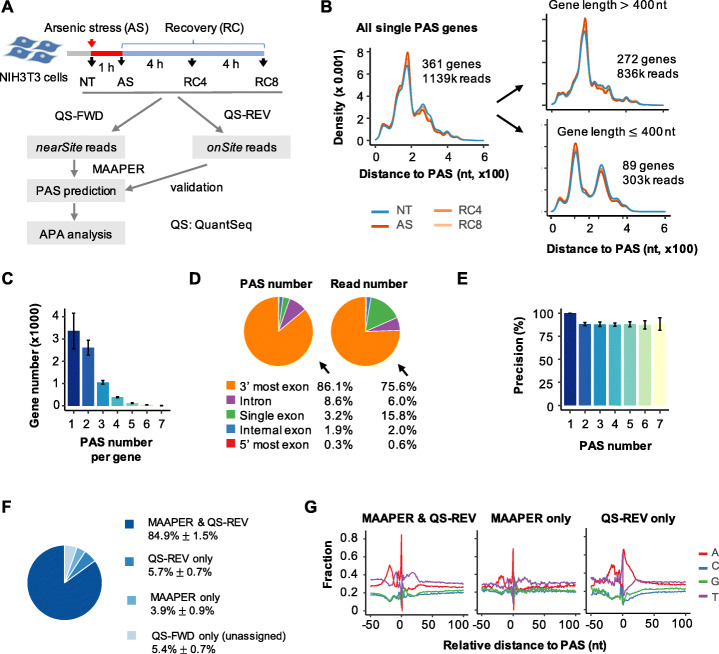
Accurate and sensitive PAS prediction by MAAPER. **A** Schematic of experimental design using QuantSeq FWD (QS-FWD) and QuantSeq REV (QS-REV) to examine APA in NIH3T3 cells treated with arsenic stress (AS) or undergoing recovery for 4 h (RC4) or 8 h (RC8) after AS. NT, non-treated cells. **B** Left, distribution of read-PAS distances based on genes with a single PAS. Right, as in the left plot, except that genes are divided into >400 nt (top) and ≤400 nt (bottom) groups. **C** Number of genes with a varying number of predicted PASs by MAAPER from the QS-FWD data. Bars denote the average gene number across all samples, and error bars denote SD. **D** Proportions of predicted PASs and QS-FWD reads based on their genomic positions. Proportions are averaged across all samples. **E** Precision rates of MAAPER’s PAS prediction for genes with a varying number of PASs. Bars denote the average precision across all samples. Error bars denote SD. **F** Proportions of four types of reads based on PAS prediction by MAAPER as well PAS identification by using QS-REV data. Mean and SD are based on all samples. **G** Nucleotide frequencies around three types of predicted PASs listed in **F**

To aid in MAAPER development, we used the QuantSeq FWD method to generate nearSite read data from mouse NIH3T3 cells under four conditions, namely, nontreated cells (NT), cells treated with arsenite stress (AS), and cells recovered from AS treatment for 4 or 8 h (RC4 or RC8, respectively) (Fig. 2A, see the “Materials and methods” section for detail). We chose these four conditions because we previously found substantial APA changes across these conditions [20]. We generated ~28 million (M) mapped reads per sample (Additional file 1: Table S1). For comparison and validation, we subjected the same RNA samples to sequencing by QuantSeq REV, a method that generates onSite reads. Importantly, QuantSeq FWD and REV methods have the same protocols except for the sequencing step, allowing us to compare nearSite reads and onSite reads more accurately without technical noise coming from variations in sample acquisition or processing.

Using the nearSite reads from QuantSeq FWD, we detected 7870 genes on average from all four samples. Of these, 361 were annotated as single-PAS genes in the PolyA_DB database [18], which we then focused on for modeling of the read-PAS distance. Interestingly, we found that read-PAS distance had two modes, one centered around 190 nucleotides (nt) from the PAS and another 250 nt from the PAS. Further analysis indicated that the 250-nt peak came from short genes (≤400 nt) (Fig. 2B). Therefore, we only used the first peak to represent read-PAS distribution in our analysis.

On average, 44.3% of the genes had a single PAS with detectable expression in the four samples and 34.4%, 13.9%, and 7.4% had 2, 3, and >3 PASs detected, respectively (Fig. 2C). These numbers are consistent across all four samples, with a standard deviation (SD) of 3.9%, 1.1%, 1.3%, and 0.4%, respectively. Of all the predicted PASs, 89.3% were in the 3′-most exons (including single-exon genes) and 8.6% were in introns (Fig. 2D, left), corresponding to 91.4% and 6.0%, respectively, of all read numbers (Fig. 2D, right).

We next used the onSite reads from QuantSeq REV to validate the predicted PASs, based on the 3′-most position of each read. Because data were generated from the same samples, the onSite reads practically served as an *ad hoc* PAS database in the same biological context, in lieu of PolyA_DB. With the 11 M mapped onSite reads from QuantSeq REV (Additional file 1: Table S1), we identified ~16 thousand (K) PASs in the four samples. Using these PASs, we calculated precision and recall rates, with precision being defined as the percentage of PASs identified by nearSite reads that matched those by onSite reads, and recall as the percentage of PASs identified by onSite reads that matched those by nearSite reads. MAAPER achieved an overall precision of 90.7% (SD = 2.0%) across the four samples, and 87.2–100% for genes with different numbers of PAS (Fig. 2E); the overall recall for all genes was 75.5% (SD = 2.5%), and 81.9% (SD = 1.7%) for genes with ≤3 PASs (Additional file 1: Figure S3A-C).

We found that the PASs identified by both onSite reads and MAAPER (“MAAPER & QS-REV,” 84.9% of nearSite reads) and PASs that were identified by MAAPER only (“MAAPER only,” 3.9% of nearSite reads) showed similar nucleotide profiles around the PASs (Fig. 2F, G and Additional file 1: Figure S4), with upstream A-rich and downstream U-rich peaks that are characteristic of PASs [21]. By contrast, the PASs identified by onSite reads but not by MAAPER (“QS-REV only,” 5.7% of nearSite reads) showed A-rich peaks both in upstream and downstream regions (Fig. 2G and Additional file 1: Figure S4). These results indicate that “QS-REV only” reads were likely to have been generated by priming of oligo(dT) at internal A-rich sequences instead at the poly(A) tail, a problem known as *internal priming*. The internal priming issue is commonly associated with methods using oligo(dT) for cDNA construction [22], but is well addressed in methods that do not depend on oligo(dT) for making cDNAs, such as 3′READS [19]. Because PolyA_DB database v3 contains 3′READS-mapped PASs only, the PASs predicted by MAAPER are not affected by the internal priming issue (Fig. 2G). As expected, MAAPER achieved an even higher overall recall of 86.6% (SD = 2.0%) after the “QS-REV only” reads were excluded (Additional file 1: Figure S3D-F).

### Differential APA analysis by MAAPER

**Fig. 3.**
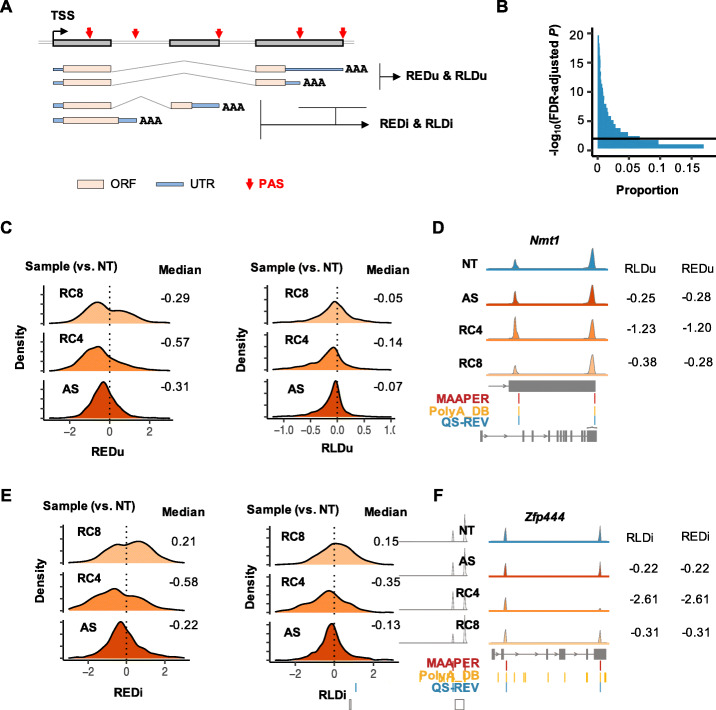
Differential APA analysis by MAAPER using QuantSeq FWD data. **A** Schematic illustrating the calculation of relative expression difference (RED) and relative length difference (RLD) scores. REDu and RLDu are for 3′UTR APA, whereas REDi and RLDi are for intronic APA. ORF, open reading frame. **B** Distribution of -log_10_(FDR-adjusted *P*) in differential APA analysis. The plotted data are based on the average across all samples. The adjusted *P* = 0.01 was used as a threshold to identify significant APA events. **C** Distributions of REDu (left) and RLDu (right) scores calculated for AS, RC4, and RC8 samples compared with NT. Only genes that showed significant APA changes in at least one sample were included. **D** Read coverage and PAS prediction results for the gene *Nmt1.* **E** Distributions of REDi (left) and RLDi (right) scores calculated for AS, RC4, and RC8 samples compared with NT. Only genes that showed significant APA changes in at least one sample were included. **F** Read coverage and PAS prediction results for the gene *Zfp444*

MAAPER examines two types of APA, i.e., 3′UTR APA and intronic APA (illustrated in Fig. 3A), separately. MAAPER produces REDu and RLDu scores for 3′UTR APA, and REDi and RLDi scores for intronic APA. REDu measures the relative expression levels between the top two most differentially expressed isoforms in the 3′-most exon only, and RLDu measures the relative 3′UTR size change based on all PASs in the 3′-most exon. By contrast, REDi measures the relative expression levels between the top differentially expressed isoform in the 3′-most exon and the top differentially expressed isoform in an intron or middle exon. RLDi measures the relative pre-mRNA size change when there are intronic PASs (all PASs upstream of the last exon are included, see the “Materials and methods” section for detail).

Using FDR-adjusted *P* value = 0.01 as the threshold, we identified significant APA events in AS, RC4, or RC8 samples compared with the NT sample (Fig. 3B). In line with our previous findings [20], AS, RC4, and RC8 samples all displayed preferential expression of isoforms using proximal PASs compared with distal PASs in 3′UTRs (median REDu = − 0.31, − 0.57, and − 0.29, respectively, Fig. 3C, left). Consistently, RLDu values indicated that 3′UTR length overall shortened in all samples after stress, with the RC4 sample showing the greatest extent (median RLDu = − 0.07, − 0.14, and − 0.05 for AS, RC4, and RC8, respectively, Fig. 3C, right). One example gene *Nmt1* is shown in Fig. 3D, and several additional ones, including *Calm1, Timp2,* and *Purb*, are shown in Additional file 1: Figure S5A. These results are in good agreement with our previous results based on 3′READS and RT-qPCR data [20].

Notably, based on REDi and RLDi, MAAPER revealed global upregulation of intronic polyadenylation (IPA) isoforms and overall pre-mRNA shortening in AS and RC4 samples (median REDi = − 0.22 and − 0.58, respectively; median RLDi = − 0.13 and − 0.35, respectively; Fig. 3E). By contrast, the RC8 sample showed the opposite trend (median REDi and RLDi = 0.21 and 0.15, respectively; Fig. 3E). One example gene *Zfp444* is shown in Fig. 3F, and several additional ones, including *Taf6, Nup155, and Elf1,* are shown in Additional file 1: Figure S5B. These results, which have not been reported before, indicate that cellular stress activates IPA, leading to shortening of pre-mRNAs.

### MAAPER enables APA analysis with single-cell data

A growing number of scRNA-seq experiments generate nearSite reads [8, 14]. To apply MAAPER to APA analysis in single cell populations, we downloaded two 10x Genomics scRNA-seq datasets for placental samples generated by Vento-Tormo et al. [23] and by Tsang et al. [24]. We were interested in placental samples because we recently found substantial APA differences among the three trophoblast cell types in placenta [25], namely, villous cytotrophoblasts (VCTs), extravillous trophoblasts (EVTs), and syncytiotrophoblasts (SCTs). The Vento-Tormo et al. dataset contained five donors at the 1st trimester [23] and the Tsang et al. dataset were from four donors at the 3rd trimester [24]. Thus, these two datasets represent trophoblasts at different stages of pregnancy.

**Fig. 4:**
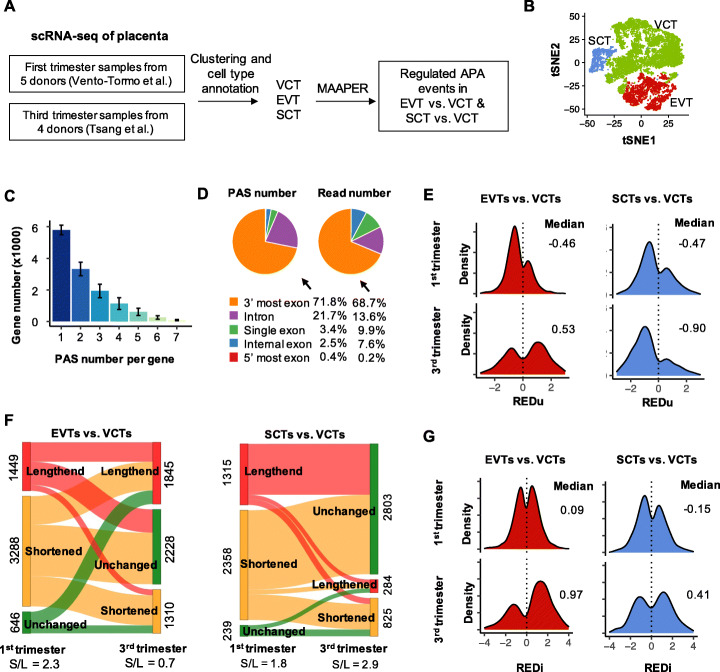
Differential APA analysis of single-cell data by MAAPER. **A** Outline of APA analysis of placental data (10x Genomics) using MAAPER. **B** tSNE plot of the three trophoblast cell types based on gene expression in the Vento-Tormo et al. data. **C** Number of genes with a varying number of predicted PASs based on the Vento-Tormo et al. data. Bars denote the average gene number across three cell types; error bars denote SD. **D** Proportions of predicted PASs and nearSite reads based on their genomic positions. Proportions were averaged across all Vento-Tormo et al. samples. **E** Distributions of REDu scores calculated for EVTs vs. VCTs (left) and SCTs vs. VCTs (right). The 1st trimester (Vento-Tormo et al.) results are shown on the top, and the 3rd trimester (Tsang et al.) results at the bottom. Only genes that showed significant APA changes in the corresponding samples are included. **F** Sankey plots showing APA changes between the two trimesters in EVTs vs. VCTs (left) or SCTs vs. VCTs (right). Gene numbers are marked on the two sides of the Sankey plots. Genes are categorized as lengthened or shortened based on REDu scores (genes without significant changes are labeled as “unchanged”). Only genes with significant changes in at least one condition, either 1st trimester or 3rd trimester, are shown. S/L = no. of genes showing 3′UTR shortening/no. of genes showing 3′UTR lengthening. **G** As in **E**, except that REDi scores are shown

We first isolated cells that belonged to VCTs, EVTs, and SCTs in each sample using cell type labels reported in the original publications (Fig. 4A, B and Figure S6). We then used MAAPER to identify PASs. For the Vento-Tormo et al. dataset, MAAPER on average identified 12,674 expressed genes in the three trophoblast cell populations, among which 44.0%, 25.2%, 14.7%, and 16.1% had 1, 2, 3, and >3 PASs, respectively (Fig. 4C). Overall, 75.2% of predicted PASs were in the 3′-most exons (including single-exon genes) and 21.7% were in introns, accounting for 78.6% and 13.6% of all reads, respectively (Fig. 4D).

Using FDR-adjusted *P* = 0.01 as the threshold, MAAPER identified significant APA changes in 6382 genes between EVTs and VCTs and 4796 genes between SCTs and VCTs in the 1st trimester (Vento-Tormo et al. data). The averaged median REDu across the five donors was − 0.46 for EVTs vs. VCTs and − 0.47 for SCTs vs. VCTs, indicating 3′UTR shortening in both EVTs and SCTs (Fig. 4E). Interestingly, in the 3rd trimester (Tsang et al. data), while SCTs showed greater 3′UTR shortening (median REDu = − 0.90, Fig. 4E), EVTs displayed global 3′UTR lengthening (median REDu = 0.53; Fig. 4E). Consistent with RED results, the ratios of gene number for genes showing 3′UTR shortening to those showing 3′UTR lengthening (or S/L) were 1.8 and 2.9 for SCTs (vs. VCTs) in 1st and 3rd trimesters, respectively (Fig. 4F). By contrast, they were 2.3 and 0.7 for EVTs (vs. VCTs) in 1st and 3rd trimesters, respectively (Fig. 4F).

In line with 3′UTR APA results, EVTs showed greater REDi score differences between the two trimesters (median REDi = 0.09 and 0.97 for 1st and 3rd trimester samples, respectively) than did SCTs (median REDi = −0.15 and 0.41 for 1st and 3rd trimester samples, respectively) (Fig. 4G). Consistently, Gene Ontology (GO) analysis of gene expression changes between the two trimesters showed more significant GO terms associated with EVTs than with SCTs (Additional file 1: Figure S7). Taken together, our MAAPER analysis results of placental single-cell data not only confirmed previous finding of 3′UTR shortening in SCT differentiation in placenta [25], but also revealed dynamics of APA changes in both EVTs and SCTs during pregnancy.

### MAAPER conducts both unpaired and paired tests.

**Fig. 5:**
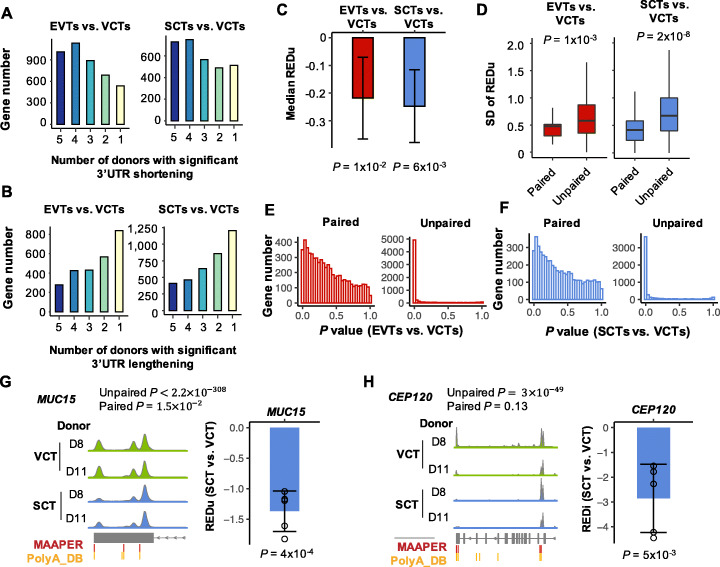
Single-cell analysis by MAAPER using a paired test. **A** Number of genes with significant 3′UTR shortening in EVTs vs. VCTs (left) or SCTs vs. VCTs (right) for the number of donors showing significant regulation. **B** As in **A** except that number of genes with significant 3′UTR lengthening is shown. **C** Median REDu scores calculated for SCTs vs. VCTs and EVTs vs. VCTs (Vento-Tormo et al. data) across five individuals. Only genes that showed significant APA changes in corresponding samples are included for plotting. Error bars indicate the SD of median values across donors, and *P* values are based on the one-sided t tests. **D** SD of REDu scores using significant genes identified by paired or unpaired tests for EVTs vs. VCTs (left) or SCTs vs. VCTs (right). FDR-adjusted *P* value = 0.1 is used as a threshold to select significant genes. Displayed *P* values are based on the Wilcoxon rank sum tests. **E**, **F** *P*-value distributions in paired or unpaired tests for EVTs vs. VCTs (**E**) or SCTs vs. VCTs (**F**). **G**, **H** Example genes *MUC15* (**G**) and *CEP120* (**H**). Aggregated reads from two donors (D8 and D11) are displayed. *P* values of RED scores are based on the one-sided t tests

While EVTs and SCTs both showed global 3′UTR shortening compared to VCTs in the 1st trimester, some genes showed 3′UTR lengthening (Fig. 4E). Using the Vento-Tormo et al. dataset, which contained five donors, we found that whereas the number of genes showing 3′UTR shortening increased along with the number of donors having significant APA regulation in both EVTs and SCTs (Fig. 5A), an opposite trend was observed for genes showing 3′UTR lengthening (Fig. 5B). This result indicates that 3′UTR shortening is more reliable than 3′UTR lengthening across donors, and APA regulation can differ across individuals.

To more directly address individual differences in APA, we used the paired test function in MAAPER, by which EVTs or SCTs were compared to VCTs from the same donor. MAAPER uses an *F* test to account for the correlation structure and to detect consistent APA changes between samples/individuals (see the “Materials and methods” section for detail). As shown in Fig. 5C, while both EVTs and SCTs showed a negative median REDu value (median across genes) averaged over the five donors, indicating 3′UTR shortening, the error bars were quite large. Indeed, analysis of standard deviation (SD) across five donors indicated that unpaired tests contained higher data variability than paired tests (Fig. 5D). As expected, paired-tests overall resulted in larger *P* values than did unpaired tests (Fig. 5E, F).

To illustrate individual differences in APA, we show two example genes, *MUC15* and *CEP120*. *MUC15* displayed significant 3′UTR shortening in SCTs vs. VCTs based on both unpaired and paired tests (Fig. 5G and Additional file 1: Figure S8A), indicating individual consistency. By contrast, *CEP120* displayed significant 3′UTR shortening in SCTs vs. VCTs based on unpaired test only but not paired test (Fig. 5G and Additional file 1: Figure S8B), indicating individual variation. Two additional example genes, *ETNK1* and *PATJ*, similar to *MUC15* and *CEP120*, respectively, are shown in Additional file 1: Figure S9. Together, our data indicate that individual APA variations can be well addressed by the paired test function of MAAPER.

### MAAPER improves accuracy of APA analysis with single-cell data

To further evaluate the applicability and performance of MAAPER in single-cell analysis, we compared our method with two other APA analysis methods that were specifically developed for single-cell data, scAPA [17] and Sierra [16]. MAAPER is distinct in several aspects from these two programs (summarized in Table 1). First, due to the use of PolyA_DB, MAAPER identifies PAS locations with single-nucleotide resolution, while scAPA and Sierra, which conduct de novo PAS prediction, provide genomic ranges with predicted peaks. Second, while all three methods perform differential analysis using single-cell clusters, only MAAPER offers paired test, taking into consideration inter-sample/individual variation. Notable also is that both PAS prediction and APA analysis in MAAPER are based on cell clusters, whereas Sierra and scAPA identifies PASs in single cells (Table 1).

**Table 1:** Comparison of various features of MAAPER, scAPA, and Sierra

	MAAPER	scAPA	Sierra
Input data type	Bulk RNA-seq or scRNA-seq	scRNA-seq	scRNA-seq
Applicable to multiple samples	Yes	Yes	Yes
PAS resolution	Individual PAS	Range based on read peak	Range based on read peak
PAS identification method	Modeling based on known PASs	De novo	Do novo
Basis for PAS quantification	Cell cluster	Single cell	Single cell
Basis for APA analysis	Cell cluster	Cell cluster	Cell cluster
Individual-based paired test	Yes	No	No

**Fig. 6:**
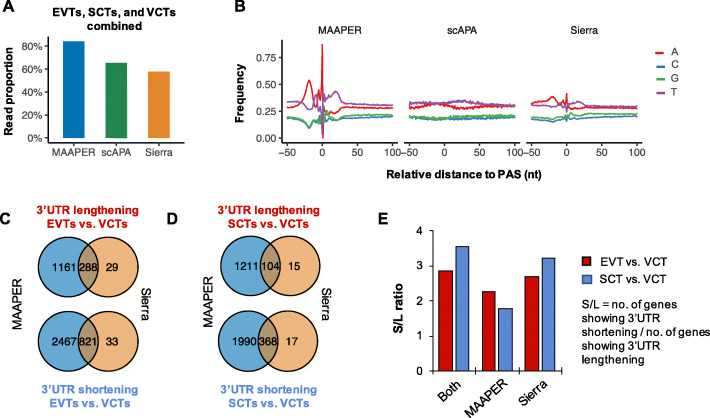
Comparisons of MAAPER, scAPA, and Sierra. **A** Proportion of scRNA-seq reads explained by the predicted PASs of the three methods. The Vento-Tormo et al. data were used. Data from EVTs, SCTs, and VCTs were combined. **B** Nucleotide frequencies around predicted PASs by the three methods. Data from EVTs, SCTs, and VCTs were combined. **C** Venn diagrams showing overlaps of significant genes with 3′UTR lengthening or shortening in EVTs vs. VCTs as identified by MAAPER and Sierra. **D** As in **C**, except that SCTs vs. VCTs data are shown. **E** S/L ratios calculated using significant genes identified by both MAAPER and Sierra, by only MAAPER, or by only Sierra

To quantitatively compare method performance, we applied scAPA and Sierra to the same 10x Genomics data of 1st trimester placental samples we used in the above analyses [23]. MAAPER identified the highest number of genes (12,674) and PASs (31,850), which were comparable with those by Sierra (11,591 genes and 28,093 PASs). By contrast, scAPA had a much lower detection rate (4979 genes and 5479 PASs) (Figure S10A-B). In addition, 84% of the reads can be assigned by MAAPER to its identified PASs, while scAPA and Sierra assigned 65.3% and 57.7%, respectively (Fig. 6A). Importantly, we found that the PASs predicted by MAAPER had discrete upstream A-rich and downstream U-rich peaks that are characteristic of genuine PASs (Fig. 6B) [26]. By contrast, these peaks were less distinct for Sierra-predicted PASs and were almost not discernable for scAPA-predicted ones (Fig. 6B). Taken together, these data indicate that MAAPER is more sensitive and accurate in PAS identification than Sierra and scAPA.

Next, we compared significant APA events identified by the three methods. With a universal threshold of FDR-adjusted *P* = 0.01, scAPA identified only <50 significant APA events in both EVTs and SCTs whereas MAAPER and Sierra on average reported 5589 and 1214 significant APA events, respectively. This result indicates that MAAPER is the most sensitive method for APA analysis among the three methods. We found that, in both SCTs and EVTs, >87% of genes with significant 3′UTR changes identified by Sierra were also detected by MAAPER (Fig. 6C, D). Interestingly, the events identified by both methods or by MAAPER only showed similar REDu scores, while events identified by Sierra only had smaller absolute REDu values (Additional file 1: Figure S10C), indicating that MAAPER is more selective than Sierra.

Based on the ratio of number of genes showing 3′UTR shortening to genes showing 3′UTR lengthening, or S/L, we found that MAAPER and Sierra reported S/L = 2.3 and 2.7, respectively, for EVTs vs. VCTs, and S/L = 1.8 and 3.2, respectively, for SCTs vs. VCTs (Fig. 6E). Using commonly regulated events, the S/L values increased to 2.9 and 3.5 for EVTs vs. VCTs and for SCTs vs. VCTs, respectively (Fig. 6E), indicating that combining the two methods could make the overall APA direction even more obvious.

Lastly, we compared the computational efficiency of the three methods using a computer with the Ubuntu 16.04.5 operating system and Intel Xeon “Gold” 6230R processor (16 cores). MAAPER on average had 12.9% less memory usage, but took 112% more time compared with Sierra (Additional file 1: Table S2). scAPA showed the lowest performance among the three, with the most memory usage and time consumption (Additional file 1: Table S2).

## Discussion

Here we present MAAPER, a computational tool that predicts PASs and conducts APA analysis using nearSite reads from bulk RNA-seq or scRNA-seq data. By incorporating well-annotated PASs in the PolyA_DB database [18], MAAPER identifies PASs located in 3'UTRs and introns with high accuracy and sensitivity. With a rigorous statistical framework based on a likelihood model, MAAPER provides statistically interpretable results and multiple metrics evaluating APA changes. In addition, paired APA analysis, first in its class to the best of our knowledge, identifies common and distinct APA events in cell populations from multiple individuals.

We applied MAAPER to QuantSeq FWD and 10x Genomics data in this work, showing that MAAPER can be used for analysis of both bulk RNA-seq and scRNA-seq data. While RNA-seq by QuantSeq FWD is a commonly used method, the volume of scRNA-seq data is increasing much more rapidly in recent years. We could therefore imagine that the application of MAAPER to scRNA-seq data will be more common than bulk RNA-seq data in the future.

MAAPER is an annotation-assisted method. It incorporates the large set of annotated PASs in the PolyA_DB database for PAS prediction. We show that this approach ensures data quality and achieves both high precision and recall. On the other hand, dependence on PolyA_DB limits the application of MAAPER. First, only the four species currently available in the database can be analyzed, including human, mouse, rat, and chicken. Second, PASs that have not been annotated by PolyA_DB, e.g., those utilized in specialized cells or under specific conditions, are not used in PAS prediction by MAAPER. One remedy is to extend the PAS prediction to novel sites. Alternatively, using additional PAS databases, such as PolyASite [27], can help mitigate this issue. Nevetheless, we also expect the sensitivity of MAAPER to increase when PAS coverage in PolyA_DB expands in the future.

For single-cell analysis, the current MAAPER version uses aggregated single cells of the same type and treats them as pseudo-bulk samples. This approach is in contrast to the two other methods we have compared with in this study, namely, scAPA and Sierra. Our rationale is that read counts summarized from scRNA-seq data often contain missing values due to biological and technical reasons [28]. As such, single cell-based APA analysis could have high noise levels [29]. With improvement in sensitivity of scRNA-seq methods, one future direction for MAAPER is to incorporate statistical tests for APA changes in individual cells.

Single cells from a given sample are naturally paired by sample identity. The paired test in MAAPER is useful in revealing cell-to-cell differences and distinctions among individuals. In this study, we show distinct cell populations in placenta could be compared within each individual and the differences across individuals could be analyzed to derive inter-individual variation. One limitation in this study is the number of individuals available for analysis, which negatively impacts the statistical power. We expect the paired test to have higher power as more individuals are included in analysis.

APA changes could provide additional information to gene-based analysis to distinguish cell types. A case in point is our finding that 3′UTR sizes differ substantially between first and third trimesters in EVTs, which is also coupled with global gene expression changes. By contrast, while gene expression changes also take place between first and third trimesters in SCTs, the 3′UTR size appears more consistent between the two trimesters. These results are in line with the notion that trophoblast cells at different stages of pregnancy have different biological characteristics, including invasiveness and proliferative activity in different trimesters [30]. Further, our results highlight the dynamic nature of APA in different cells under distinct conditions, supporting their value in defining cell identity.

## Materials and methods

### Cell culture and RNA sequencing

NIH3T3 cells were cultured in high glucose Dulbecco’s modified Eagle’s medium (DMEM) supplemented with 10% calf serum, 100 IU/ml penicillin, and 100 μg/ml streptomycin at 37 °C. For stress induction, cells were cultured in medium containing 250 μM of sodium arsenite (NaASO_2_) for 1 h. For recovery, stressed cells were washed twice with phosphate-buffered saline (PBS) to remove NaASO_2_, followed by culturing in regular medium for 4 h or 8 h. Total RNA was extracted using TRIzol. QuantSeq 3′ mRNA-Seq Library Prep Kits (FWD or REV) were used to prepare libraries. Sequencing was carried out on an Illumina NextSeq.

### Summary of the MAAPER method

MAAPER is a novel method for predicting PAS locations and comparing APA usage using nearSite reads, and it has four unique features which contribute to its high accuracy and broad applicability. First, the statistical model of MAAPER is flexible. Unlike most existing PAS prediction tools (develop for RNA-seq) that use a simplified model of only two PASs (proximal and distal), MAAPER predicts a comprehensive set of expressed PASs for each gene, including those located in 3'UTRs, internal exons, and introns. Second, MAAPER has high accuracy in PAS prediction. On the one hand, it achieves a high recall rate by incorporating the large collection of PASs annotated in PolyA_DB database, which contains more than 85,000 and 120,000 PASs for human and mouse genes in its current version (v3). On the other, it achieves a high precision by a forward-selection procedure, which screens for a conservative set of PASs that adequately explain the observed reads. Third, MAAPER is statistically principled. It detects differential PAS usage between sample groups using rigorous and flexible statistical tests. Most existing methods for RNA-seq or onSite reads use the Fisher’s exact test [31] for differential PAS usage, which is limited to comparisons between two samples and two PASs [4–7]. In contrast, for unpaired samples, MAAPER formulates an LRT based on its likelihood model to accurately detect if a gene’s PAS proportions change between conditions; for paired samples, MAAPER uses an *F*-test to account for the correlation structures and detects consistent APA changes between conditions. Fourth, MAAPER facilitates the interpretation of differential analysis of PAS usage. In addition to estimated PAS proportions, it calculates two scores to measure the differences in relative transcript length and relative PAS expression, providing a convenient approach to quantifying APA regulation.

### Likelihood model for PAS prediction

MAAPER uses a rigorous and flexible likelihood-based framework to identify PASs and quantify APA isoform abundance with 3′ end-linked RNA sequencing data (nearSite reads). There are two steps at the PAS prediction stage. At the first step, MAAPER learns the distance of nearSite reads to PASs using only genes with a single PAS. At the second step, MAAPER uses a likelihood model to predict PASs for all reads. MAAPER uses data from the PolyA_DB database (v3), a comprehensive PAS database based on 3′READS data [18], at both steps. Specifically, for each sample, after aligning reads to the reference genome, we extract all the reads mapped to the single PAS genes (annotated in PolyA_DB) and calculate the distances (excluding intronic regions) between the 5′ end positions of these reads and their corresponding PASs. Next, we non-parametrically estimate the probability density of the distances using a Gaussian kernel function. This is achieved with the density function in R. This approach allows us to capture the distributional characteristic of the distances without assuming uniformness, which is often violated in real data [32]. In our analysis, we learned distances for genes ≤400 nt and genes >400 nt separately, as we have found that the distances for short genes displayed different distributional properties (Fig. 2B).

At the second step, MAAPER constructs a likelihood model to identify PASs and quantify isoform abundance. Suppose a given gene has *J* annotated PASs in PolyA_DB, then we denote their proportions in the mRNAs transcribed from this gene as ${\alpha}_1,\dots, {\alpha}_J\ \left({\sum}_{j=1}^J{\alpha}_j=1\right).$ Note that the model aims to detect all PASs in all samples. For sample replicates, we denote the replicate number as *K*_1_ in condition 1 and as *K*_2_ in condition 2. When there is no replicate, *K*_1_ = *K*_2_ = 1. In addition, we use *n*_*ck*_ to denote the number of reads mapped to the gene in the *k*-th replicate in condition *c* (*c* = 1 or 2). Given the above notations, the joint likelihood of nearSite reads mapped to this gene can be derived as

$$ L={\prod}_{c=1}^2{\prod}_{k=1}^{K_c}{\prod}_{i=1}^{n_{ck}}{\sum}_{j=1}^J{\left({P}_{ck i j}{\alpha}_j\right)}^{\mathbbm{I}\left\{{Z}_{ck i}=j\right\}}, $$

where *Z*_*cki*_ ∈ {1, …, *J*} is the unobserved PAS index and *Z*_*cki*_ = *j* if the *i*-th read in the *k*-th replicate in condition *c* comes from a transcript using the *j*-th PAS. *P*_*ckij*_ denotes the probability of observing a read at read *i*’s 5′ position in the *k*-th replicate in condition *c*, given that the read comes from a transcript using the *j*-th PAS; *P*_*ckij*_ is calculated based on the distance densities learned from the first step.

Given the above likelihood function, we developed an Expectation-Maximization (EM) algorithm [33] to maximize the log-likelihood

$$ l={\sum}_{c=1}^2{\sum}_{k=1}^{K_c}{\sum}_{i=1}^{n_{ck}}{\sum}_{j=1}^J\mathbbm{I}\left\{{Z}_{ck i}=j\right\}\log \left({P}_{ck i j}{\alpha}_j\right), $$

and estimate the PAS proportions. We use ${\alpha}_j^{(0)},\dots, {\alpha}_J^{(0)}$ to denote initial values and use ${\alpha}_j^{(t)},\dots, {\alpha}_J^{(t)}$ to denote estimated values in the *t*-th iteration of the EM algorithm. Then, in the (*t* + 1)-th iteration, we can update the estimators as

$$ {\alpha}_j^{\left(t+1\right)}=\frac{1}{\sum_{c=1}^2{\sum}_{k=1}^{K_c}{n}_{ck}}{\sum}_{c=1}^2{\sum}_{k=1}^{K_c}{\sum}_{i=1}^{n_{ck}}\frac{P_{ij}{\alpha}_j^{(t)}}{\sum_{j^{\prime }=1}^J{P}_{i{j}^{\prime }}{\upalpha}_{j^{\prime}}^{(t)}}\ \left(j=1,\dots, J\right). $$

We use the above algorithm to update estimated PAS proportions until convergence.

Some genes have a large number of annotated PASs in PolyA_DB (Additional file 1: Figure S1). The above EM algorithm would give most PASs non-zero proportions even though some isoforms have a very low abundance. To address this issue, MAAPER uses a forward selection procedure to select PASs into the model based on their statistical significance. MAAPER first ranks all PASs based on their supporting read numbers and starts the selection process with the PAS with the largest read number. At each subsequent step, the likelihood ratio test is carried out to determine if addition of a PAS would significantly lead to increase of the likelihood. The process is terminated when no such an increase can be achieved. In essence, MAAPER selects a minimal set of annotated PASs that can sufficiently explain the observed nearSite reads. Our previous work has shown that a similar stepwise selection procedure is very efficient in identifying truly expressed RNA isoforms from RNA-seq data [32].

### Unpaired statistical test for differential APA profiles

With the predicted PASs, MAAPER analyzes differential APA isoform abundances between the two conditions. Suppose there are *J* PASs in a given gene. We denote their proportions in condition *c* (*c* = 1 or 2) as ${\alpha}_{c1},\dots, {\alpha}_{cJ}\ \left({\sum}_{j=1}^J{\alpha}_{cj}=1\right).$ Following the notations described above, the joint likelihood of nearSite reads mapped to the gene can be derived as

$$ L={\prod}_{c=1}^2{\prod}_{k=1}^{K_c}{\prod}_{i=1}^{n_{ck}}{\sum}_{j=1}^J{\left({P}_{ck i j}{\alpha}_{cj}\right)}^{\mathbbm{I}\left\{{Z}_{ck i}=j\right\}}. $$

Unlike tools that focus on proximal and distal PASs only, we designed a likelihood ratio test to detect differential abundances for all PASs. The null hypothesis is *H*_0_ : *α*_1*j*_ = *α*_2*j*_ (*j* = 1, …, *J*); the alternative hypothesis is *H*_*a*_ :  ∃ *j* ∈ {1, …, *J*} s. t. *α*_1*j*_ ≠ *α*_2*j*_. Under *H*_0_, the proportions can be estimated using the EM algorithm described above, and we denote the estimated values after convergence as ${\hat{\alpha}}_1^0,\dots, {\hat{\alpha}}_J^0$. Under *H*_*a*_, we can also use an EM algorithm to update the estimators using the following rule:

$$ {\alpha}_{cj}^{\left(t+1\right)}=\frac{1}{\sum_{k=1}^{K_c}{n}_{ck}}{\sum}_{k=1}^{K_c}{\sum}_{i=1}^{n_{ck}}\frac{P_{ij}{\alpha}_j^{(t)}}{\sum_{j^{\prime }=1}^J{P}_{i{j}^{\prime }}{\alpha}_{j^{\prime}}^{(t)}}\ \left(c=1,2;j=1,\dots, J\right). $$

We denote the estimated values after convergence as ${\hat{\alpha}}_{c1}^a,\dots, {\hat{\alpha}}_{cJ}^a$. Under *H*_0_, the statistic below follows a chi-square distribution:

$$ -2\log \frac{L\left({\alpha}_{1j}={\alpha}_{2j}={\hat{\alpha}}_1^0,\dots, {\alpha}_{1J}={\alpha}_{2J}={\hat{\alpha}}_J^0\right)}{L\left({\alpha}_{1j}={\hat{\alpha}}_{11}^a,{\alpha}_{2j}={\hat{\alpha}}_{21}^a,\dots, {\alpha}_{1J}={\hat{\alpha}}_{1J}^a,{\alpha}_{2J}={\hat{\alpha}}_{2J}^a\right)}\sim {\chi}^2(J). $$

The *P* values of the likelihood ratio tests can then be obtained based on the above test statistic and are adjusted by the Benjamini and Hochberg method [34] to correct for the multiple testing issue and derive false discovery rate (FDR). In our analysis, we use the adjusted *P* value = 0.01 as the threshold for identifying genes with significantly differential APA isoform abundances. In addition to the LRT, MAAPER also provides the Fisher’s exact test to examine if the differences are significant for the two PASs with the largest proportion changes. When there are replicates, reads are first pooled from the replicates to carry out the Fisher’s test.

### Paired statistical test for differential APA profiles

In scRNA-seq experiments, comparison of APA profiles is often within the same group of subjects. For example, it is often of interest to test for differential APA expression between two cell types from the same subjects, or between pre- and post-treatment cells from the same subjects. To address this type of questions, we propose a test for differential APA profiles among paired subjects. Suppose there are *J* predicted PASs in a given gene. We denote their proportions in sample *i* (*i* = 1, 2, …, *n*) in condition *c* (*c* = 1 or 2) as ${\boldsymbol{\alpha}}_{ci}={\left({\alpha}_{ci1},\dots, {\alpha}_{ci J}\right)}^T\ \left({\sum}_{j=1}^J{\alpha}_{ci j}=1\right).$ The reads mapped to the given gene in each nearSite read sample are assigned to the PASs based on the read-PAS probabilities calculated in the prediction stage. We denote the read counts as ***X***_1*i*_ ∈ *ℕ*^* J*^ and ***X***_2*i*_ ∈ *ℕ*^* J*^, where *X*_*cij*_ is the read count of PAS *j* in sample *i* in condition *c*.

We assume that both ***X***_1*i*_ and ***X***_2*i*_ follow a Multinomial distribution

$$ {\boldsymbol{X}}_{ci}\sim \mathrm{Multinomial}\left({N}_{ci},{\boldsymbol{\alpha}}_{ci}\right), $$

where ${N}_{ci}={\sum}_{j=1}^J{X}_{ci j}$. In addition, we assume that the PAS proportions in samples of the same condition independently follow the same distribution, where

$$ E\left({\boldsymbol{\alpha}}_{ci}\right)={\boldsymbol{\uppi}}_c,\kern0.5em \mathrm{Var}\left({\boldsymbol{\alpha}}_{ci}\right)={\boldsymbol{\Sigma}}_c. $$

To account for the correlation between APA profiles of the same subject, we further assume that

$$ Cov\left({\boldsymbol{\alpha}}_{1i},{\boldsymbol{\alpha}}_{2i}\right)={\boldsymbol{\Sigma}}_{12}. $$

It follows from the above assumptions that

$$ E\left({\boldsymbol{X}}_{ci}\right)={N}_{ci}{\boldsymbol{\uppi}}_c, $$

$$ \mathrm{Var}\left({\boldsymbol{X}}_{ci}\right)={N}_{ci}\left(\mathrm{Diag}\left({\boldsymbol{\uppi}}_c\right)-{\boldsymbol{\uppi}}_c{\boldsymbol{\uppi}}_c^T\right)+{N}_{ci}\left({N}_{ci}-1\right){\boldsymbol{\Sigma}}_c, $$

$$ \mathrm{Cov}\left({\boldsymbol{X}}_{1i},{\boldsymbol{X}}_{2i}\right)={N}_{1i}{N}_{2i}{\boldsymbol{\Sigma}}_{12}. $$

In out paired test, the null hypothesis is *H*_0_ : *π*_1*j*_ = *π*_2*j*_ (*j* = 1, …, *J*); the alternative hypothesis is *H*_*a*_ :  ∃ *j* ∈ {1, …, *J*} s. t. *π*_1*j*_ ≠ *π*_2*j*_. Based on [35], we can construct the following *F* statistic to test the hypotheses

$$ F=\frac{n-d+1}{\left(n-1\right)\left(\mathrm{d}-1\right)}{\left({\hat{\boldsymbol{\pi}}}_1-{\hat{\boldsymbol{\pi}}}_2\right)}^T\boldsymbol{\Theta} \left({\hat{\boldsymbol{\pi}}}_1-{\hat{\boldsymbol{\pi}}}_2\right), $$

where **Θ** is the Moore-Penrose pseudoinverse of ${\hat{\boldsymbol{\Sigma}}}_{\hat{\pi}}$. In addition,

$$ {\hat{\boldsymbol{\Sigma}}}_{\hat{\pi}}={\sum}_{c=1}^2\left\{\frac{{\boldsymbol{S}}_c+\left({M}_c-1\right){\boldsymbol{G}}_c}{M_c\ {N}_c}+\frac{\sum_{i=1}^n{N}_{ci}^2-{N}_c}{M_c\ {N}_c^2}\left({\boldsymbol{S}}_c-{\boldsymbol{G}}_c\right)\right\}-\frac{\sum_{i=1}^n{N}_{1i}{N}_{2i}}{\ {N}_1{N}_2}\left({\hat{\boldsymbol{\Sigma}}}_{12}+{\hat{\boldsymbol{\Sigma}}}_{12}^T\right), $$

where ${\hat{\boldsymbol{\pi}}}_c={\sum}_{i=1}^n{\boldsymbol{X}}_{ci}$/${\sum}_{i=1}^n{N}_{ci},$ ${\hat{\boldsymbol{\pi}}}_{ci}={\boldsymbol{X}}_{ci}$/*N*_*ci*_, ${N}_c={\sum}_{i=1}^n{N}_{ci},$ ${M}_c=\frac{1}{\left(n-1\right){N}_c}\left({N}_c^2-{\sum}_{i=1}^n{N}_{ci}^2\right),$ and

$$ {\boldsymbol{S}}_c=\frac{1}{n-1}{\sum}_{i=1}^n{N}_{ci}\left({\hat{\boldsymbol{\pi}}}_{ci}-{\hat{\boldsymbol{\pi}}}_c\right){\left({\hat{\boldsymbol{\pi}}}_{ci}-{\hat{\boldsymbol{\pi}}}_c\right)}^T, $$

$$ {\boldsymbol{G}}_c=\frac{1}{N_c-n}{\sum}_{i=1}^n{N}_{ci}\left(\mathrm{Diag}\left({\hat{\boldsymbol{\pi}}}_{ci}\right)-{\hat{\boldsymbol{\pi}}}_{ci}{\hat{\boldsymbol{\pi}}}_{ci}^T\right), $$

$$ {\hat{\boldsymbol{\Sigma}}}_{12}=\frac{1}{n-1}{\sum}_{i=1}^n\frac{N_{1i}+{N}_{2i}}{M_1+{M}_2}\left({\hat{\boldsymbol{\pi}}}_{1i}-{\hat{\boldsymbol{\pi}}}_1\right){\left({\hat{\boldsymbol{\pi}}}_{2i}-{\hat{\boldsymbol{\pi}}}_2\right)}^T. $$

Under *H*_0_, the *F* statistic follows an asymptotic *F*-distribution with degrees with freedom *J* − 1 and *n* − *J* − 1. The *P* values can then be obtained based on the above test statistic, and are adjusted by the Benjamini and Hochberg method to correct for the multiple testing issue.

### APA regulation scores

MAAPER calculates four scores for APA regulation, namely relative length difference (RLD) for 3′UTR size difference (RLDu, considering 3′-most exon PASs only), RLD for pre-mRNA size difference (RLDi, considering internal PASs), relative expression difference (RED) for 3′UTR size difference (REDu, considering 3′-most exon PASs only), and RED for relative expression of internal PASs (REDi).

For RLDu, where we only consider 3′UTR PASs, suppose a gene has *J* 3′UTR PASs, ordered from 5′ to 3′ on the chromosome. Their proportions are denoted as *α*_11_, …, *α*_1*J*_ in condition 1 and *α*_21_, …, *α*_2*J*_ in condition 2. We use *l*_*j*_ to denote the relative 3'UTR length of the *j*-th PAS, with *l*_1_ = 1 nt for the 5′-most PAS and *l*_*j*_ (*j* > 1) is the nucleotide distance between the first and the *j*-th PASs on the 3′UTR. The RLDu score between conditions 1 and 2 is calculated as $\mathrm{RLDu}={\log}_2\left({\sum}_{j=1}^J{\alpha}_{2j}{l}_j/{\sum}_{j=1}^J{\alpha}_{1j}{l}_j\right)$. As such, RLDu measures the relative length of 3'UTR between the two conditions.

For RLDi, where we consider PASs located in introns and internal exons in addition to 3′-most exons, suppose a gene has *J* PASs, ordered from 5′ to 3′ on the chromosome. *J*_1_ is the number of PASs located in introns or internal exons and *J*_2_ is the number of PASs in the 3′-most exon (*J*_1_ + *J*_2_ = *J*). *l*_*j*_ denotes the length of the pre-mRNA transcript for the *j*-th PAS, i.e., the genomic distance between transcription start site and the *j*-th PAS. Then, the RLDi score can be calculated as $\mathrm{RLDi}={\log}_2\left({\sum}_{j={J}_1+1}^{J_1+{J}_2}{\alpha}_{2j}{l}_j/{\sum}_{j=1}^{J_1}{\alpha}_{2j}{l}_j\right)-{\log}_2\left({\sum}_{j={J}_1+1}^{J_1+{J}_2}{\alpha}_{1j}{l}_j/{\sum}_{j=1}^{J_1}{\alpha}_{1j}{l}_j\right)$. As such, RLDi measures the relative length of pre-mRNA transcripts with internal polyadenylation between the two conditions.

For the RED score, we use the two PASs with the largest proportion changes between two conditions. For REDu, we select the top two PASs in the 3′UTR; for REDi, we select one PAS from introns/internal exons and the other from 3′UTR. We refer to the two PASs as the distal PAS and proximal PAS based on their chromosomal positions relative to the transcription start site. Suppose the proportions of the proximal PAS in conditions 1 and 2 are denoted as *α*_1*p*_ and *α*_2*p*_, and the proportions of the distal PAS are denoted as *α*_1*d*_ and *α*_2*d*_. The RED score between conditions 1 and 2 is calculated as RED = log_2_(*α*_2*d*_/*α*_2*p*_) − log_2_(*α*_1*d*_/*α*_1*p*_). Therefore, a negative RED value indicates transcript shortening in condition 2 comapred with condition 1 and a positive value transcript lengthening.

### QuantSeq data analysis

The processing of QuantSeq data followed the protocol on the website of Lexogen (https://www.lexogen.com). Briefly, the adapter contamination, polyA read through, and low quality tails were first trimmed using bbmap [36]. The cleaned reads were then mapped to the RefSeq mm9 genome using STAR [37] with the parameters of “--outFilterType BySJout --outFilterMultimapNmax 20 --alignSJoverhangMin 8 --alignSJDBoverhangMin 1 --outFilterMismatchNmax 999 --outFilterMismatchNoverLmax 0.1 --alignIntronMin 20 --alignIntronMax 1000000 --alignMatesGapMax 1000000 --outSAMattributes NH HI NM MD”. MAAPER was applied to aligned QuantSeq FWD reads from NT, AS, RC4, and RC8 samples separately for PAS prediction and differential APA analysis. Only genes with at least 50 mapped reads were considered. At the PAS prediction stage, a proportion of 5% was used as the threshold to select PASs. We evaluated the accuracy of MAAPER by varying this threshold between 1 and 10%, and found that MAAPER’s precision was consistently above 85% and recall above 80%, demonstrating robustness of MAAPER (Additional file 1: Figure S11). At the APA analysis stage, an FDR-adjusted *P* value of 0.01 was used as the threshold to select significant cases. For PAS identification with QuantSeq REV data, the PAS positions were directly determined as the 3′-most positions of mapped reads.

### scRNA-seq data analysis

10x Genomics scRNA-seq data of first-trimester placental cells were generated by Vento-tormo et al. [23], which contained five donors. Reads were aligned against the hg19 human reference genome using the Cell Ranger Single-Cell Software Suite (version 3.0, 10x Genomics). Reads from three cell types (VCTs, EVT, and SCTs) were individually aggregated into pseudo-bulk bam files for each donor. Cell type annotations were obtained from Vento-tormo et al. [23]. MAAPER (v1.1.0) was then applied to the pseudo-bulk bam files for PAS prediction and differential APA analysis. Same thresholds were used as in the QuantSeq FWD data analysis. Data representation by tSNE based on gene expression levels was carried out using the R package Seurat (version 3.1.2) [38]. For method comparison, scAPA (v0.1.0) and Sierra (v0.99.24) were applied to the same bam files using default parameters.

10x Genomics scRNA-seq data of third-trimester placental cells were generated by Tsang et al. [24], which included four donors. Reads were processed in the same way as the first-trimester data. MAAPER (v1.1.0) was then applied to the pseudo-bulk bam files of VCTs, EVTs, and SCTs. For the differential expression analysis between the two trimesters, we first normalized the read counts by the library sizes of individual cells. Since the two datasets were generated from two independent studies, we calculated gene expression levels in each donor as the log2 ratio of normalized read counts between EVTs (or SCTs) and VCTs to control for technical variability. Then, the expression levels were compared using two-sided t tests and the 1000 most significant genes were used for GO enrichment analysis.

## Supplementary Information

**Additional file 1: Supplementary materials.** It includes all supplementary tables and figures.

**Additional file 2.** Review history.

## Data Availability

The MAAPER method has been implemented as an R package, which is available at Github (https://github.com/Vivianstats/MAAPER) [39]. The source code is available at the zenodo repository (DOI: 10.5281/zenodo.4760264) under the GPLv2 license [40]. QuantSeq FWD and REV datasets generated in this study have been deposited into the GEO database under the accession number GSE164958 [41]. 10x Genomics scRNA-seq data of first-trimester placental cells were generated by Vento-tormo et al. [23] and downloaded from ArrayExpress with accession number E-MTAB-6701. 10x Genomics scRNA-seq data of third-trimester placental cells were generated by Tsang et al. [24] and downloaded from the European Genome-Phenome Archive with accession number EGAS00001002449.
