# Supplementary material for: MAAPER: model-based analysis of alternative polyadenylation using 3′ end-linked reads
Source: Genome Biol. 2021 Aug 10;22:222. doi: 10.1186/s13059-021-02429-5 (PMC8356463; doi:10.1186/s13059-021-02429-5)
Supplement: Supplementary file 1 — Additional file 1: Supplementary materials. It includes all supplementary tables and figures. [file 13059_2021_2429_MOESM1_ESM.pdf]

Table S1. Read number of QuantSeq samples.

Sample	Number of QuantSeq FWD reads	Number of QuantSeq REV reads
NT	27,859,996	1,817,267
AS	27,859,632	3,928,573
RC4	28,788,156	1,529,184
RC8	27,948,416	3,605,416

Table S2. Computational time and memory usage.

Study	Read number	MAAPER		scAPA		Sierra	
		Memory	Time	Memory	Time	Memory	Time
QuantSeq (NT vs. AS)	55,719,628	29.9G	1.9h				
QuantSeq (NT vs. RC4)	56,648,152	28.8G	2.0h				
QuantSeq (NT vs. RC8)	55,808,412	28.9G	1.5h				
10x (VCT vs. SCT)	979,733,288	46.3G	15.4h	96.1G	36.6h	49.3G	8.6h
10x (VCT vs. EVT)	1,443,694,569	46.7G	24.1h	114.8G	43.7h	57.5G	9.9h

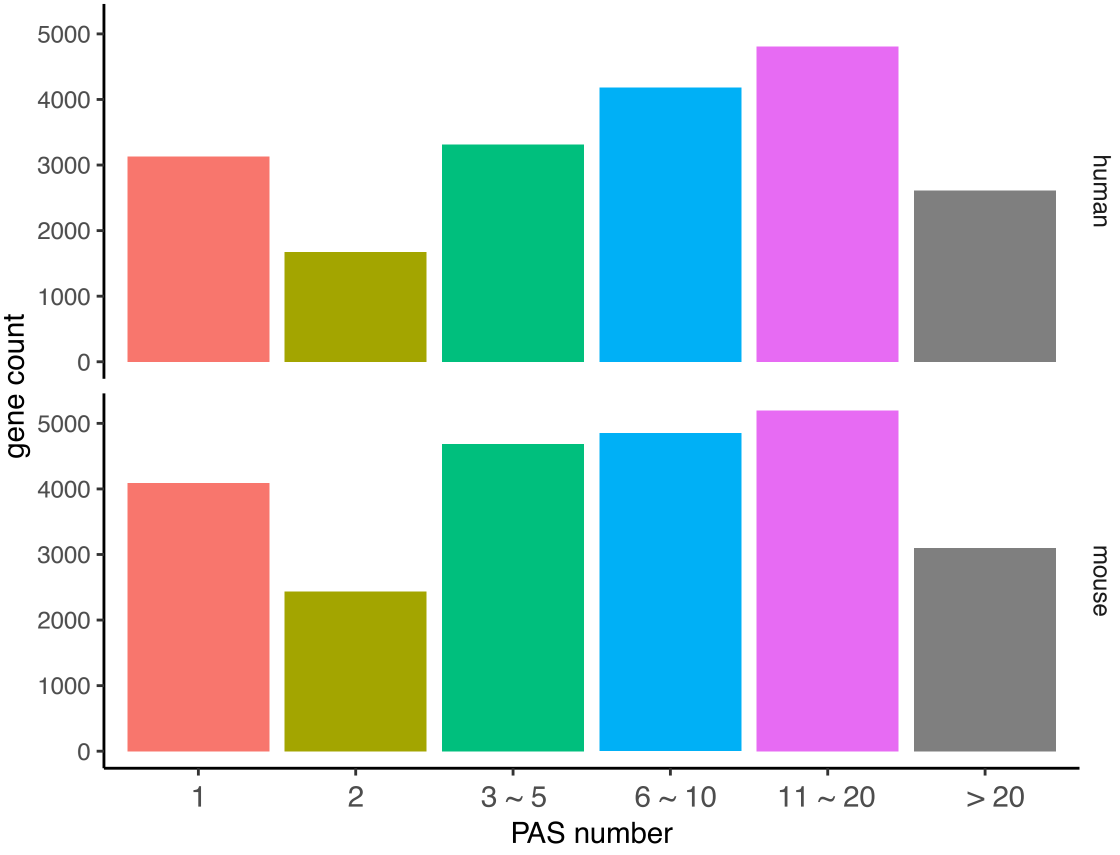

Figure S1. Number of annotated PASs in PolyA_DB. The numbers of human and mouse genes with varying number of annotated PASs are displayed as barplots. The PAS number of human genes has a median of 7 and a mean of 10.1. The PAS number of mouse genes has a median of 6 and a mean of 9.7.

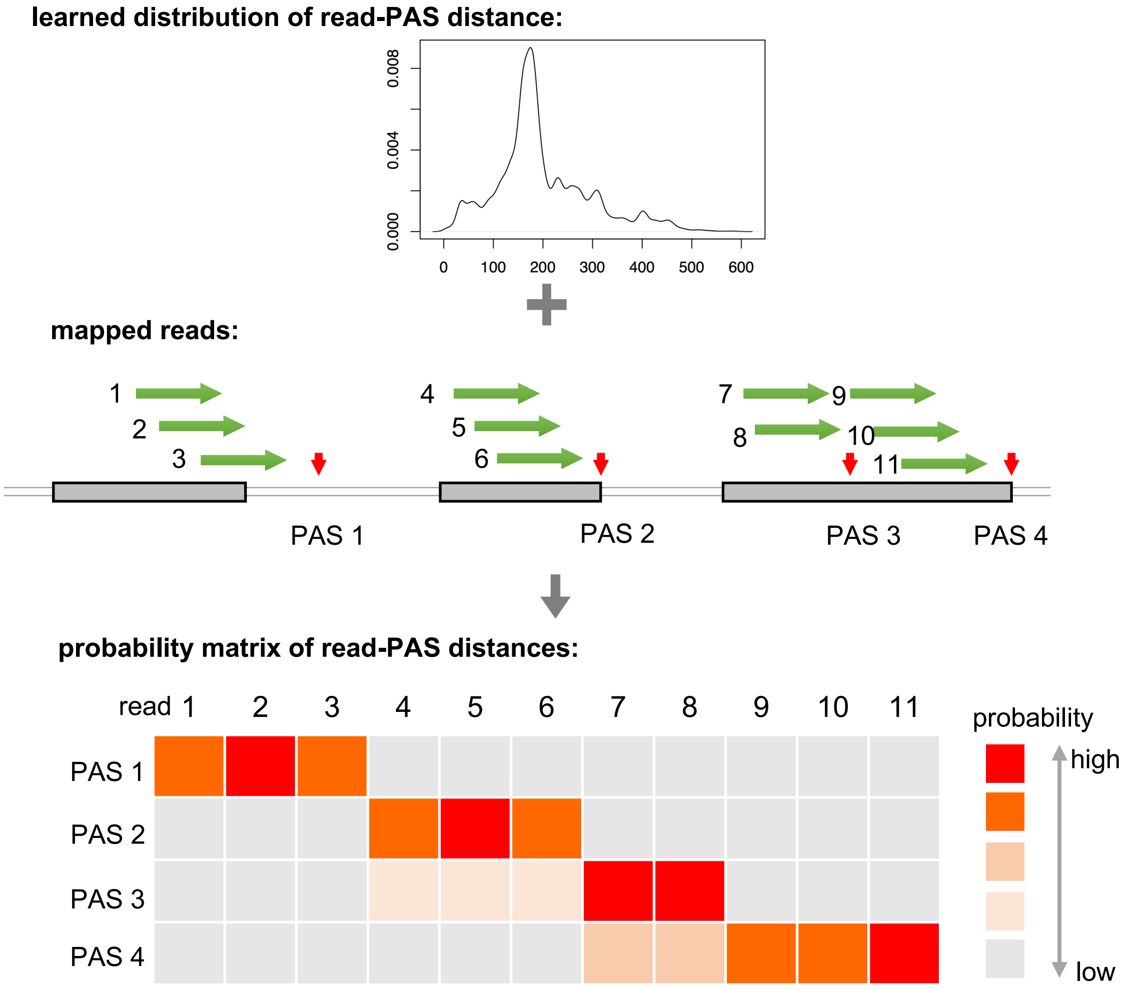

Figure S2. Illustration of the calculation of probabilities of read-PAS distances.

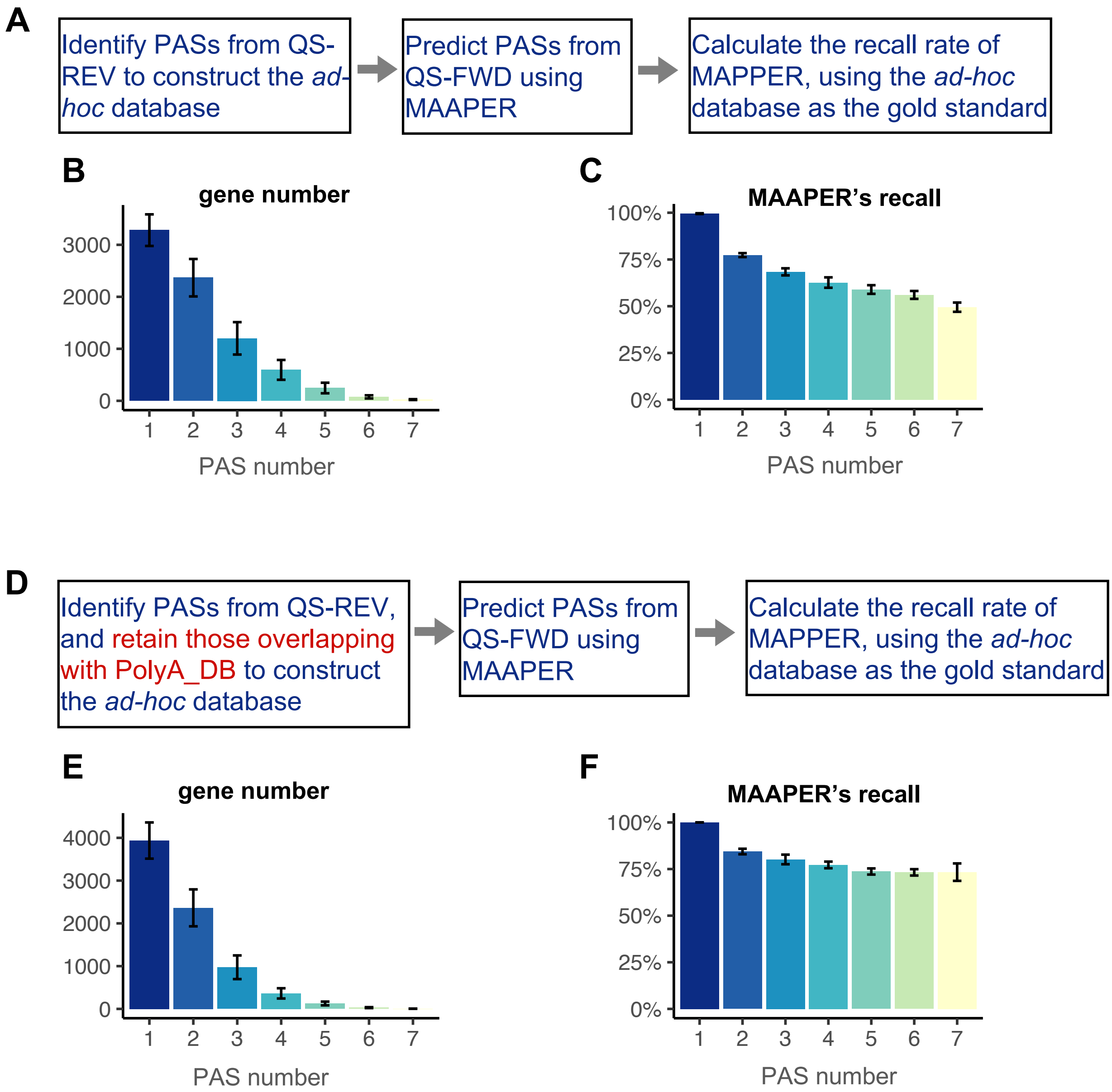

Figure S3. Recall rates of MAAPER in PAS prediction. **A-C**: Results are based on identified PASs from QS-REV data. **D-F**: Results are based on identified PASs (from QS-REV data) that are also annotated in PolyA_DB. **A,D**: Schematic diagrams showing steps to calculate MAAPER's recall rate. **B,E**: Number of genes with different number of identified PASs from QS-REV data. Gene number is averaged across NT and AS-treated conditions, and error bars represent one standard deviation. **C,F**: Recall rates of MAAPER's prediction from QS-FWD data for genes with varying number of identified PASs from QS-REV data. Recall rate is averaged across NT and AS-treated conditions, and error bars represent one standard deviation.

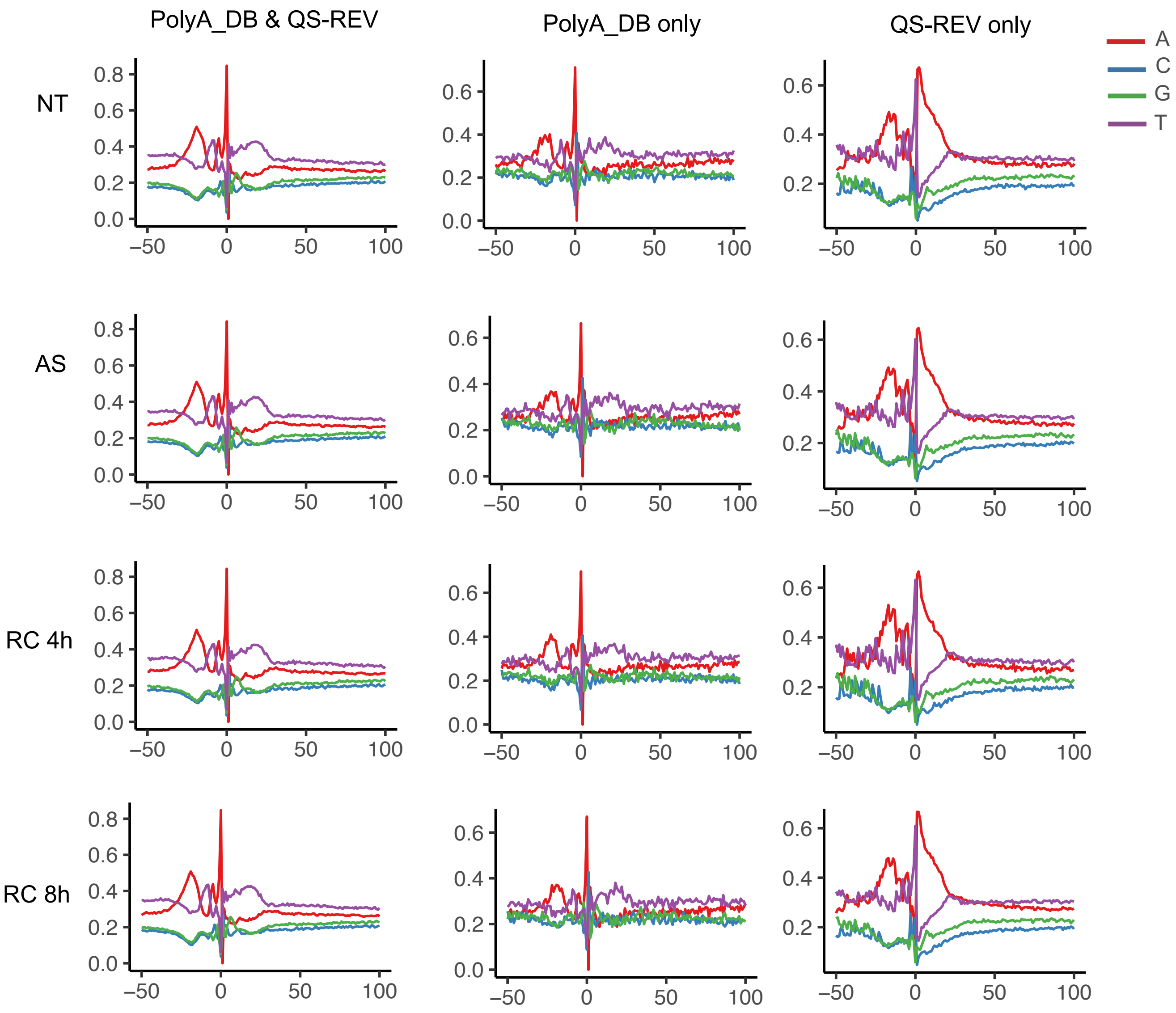

Figure S4. Nucleotide frequency around PASs. QS-FWD reads were assigned to three types of PASs: PASs predicted by MAAPER, annotated in PolyA_DB, and supported by QS-REV reads; PASs predicted by MAAPER, annotated in PolyA_DB, but not supported by QS-REV reads; PASs not annotated in PolyA_DB but supported by QS-REV reads. The average nucleotide frequency in these three types of PASs were calculated for each sample.

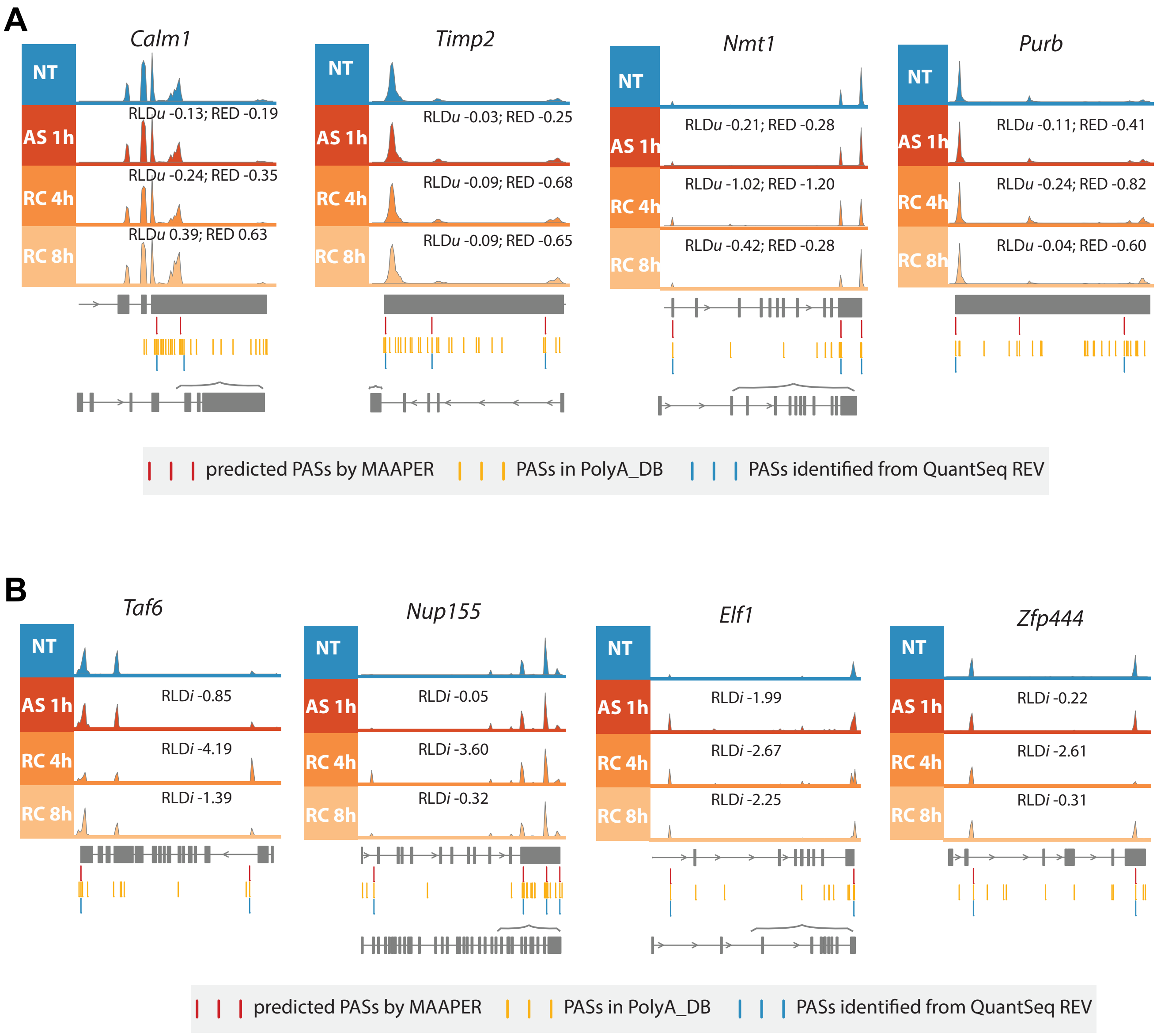

Figure S5. Read alignment tracks of example genes in NT and AS-treated samples. **A**: Four genes whose 3'UTR shortening has been validated by PCR experiments. **B**: Four genes with significant activation of intronic PASs in AS-treated conditions.

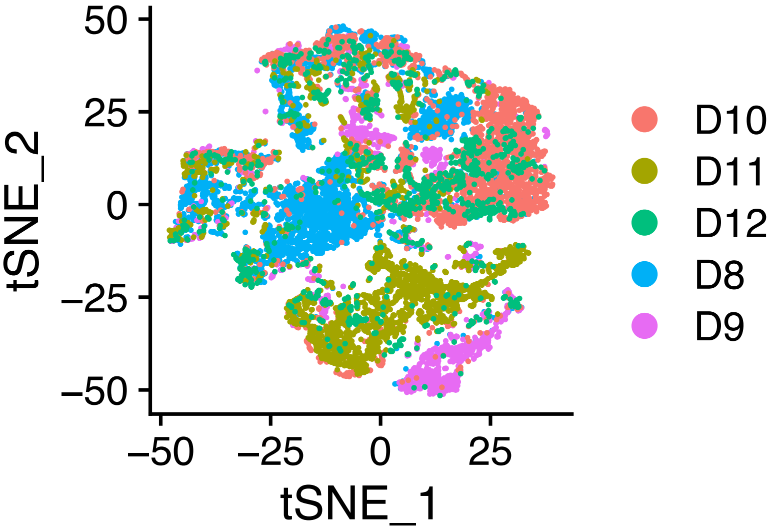

Figure S6. tSNE plots of the trophablast cells from the five donors.

A

EVT:

**Downregulated genes, 3rd vs. 1st trimester**	**Adjusted *P***
extracellular matrix organization	4.9×10^{-6}
extracellular structure organization	4.9×10^{-6}
neutrophil degranulation	2.2×10^{-5}
antigen processing and presentation	4.3×10^{-4}
regulation of actin cytoskeleton organization	5.1×10^{-4}

**Up-regulated genes, 3rd vs. 1st trimester**	**Adjusted *P***
translational termination	8.9×10^{-12}
ribonucleoprotein complex biogenesis	1.7×10^{-10}
mitochondrial translational elongation/termination	1.0×10^{-9}
ribosome biogenesis	2.3×10^{-8}
mitochondrial translation	2.3×10^{-8}

B **SCT:**

**Downregulated genes, 3rd vs. 1st trimester**	**Adjusted *P***
RNA/mRNA catabolic process	2.8×10^{-16}
protein catabolic process	3.9×10^{-13}
regulation of hematopoietic progenitor cell differentiation	9.6×10^{-10}
regulation of hematopoietic stem cell differentiation	2.8×10^{-9}
anaphase-promoting complex-dependent catabolic process	3.2×10^{-9}

**Up-regulated genes, 3rd vs. 1st trimester**	**Adjusted *P***
mitotic sister chromatid cohesion	1.6×10^{-1}
regulation of smoothened signaling pathway	1.6×10^{-1}
nuclear export	2.9×10^{-1}
negative regulation of smoothened signaling pathway	2.9×10^{-1}
pyrimidine ribonucleoside triphosphate metabolic process	2.9×10^{-1}

Figure S7. **A:** Top enriched GO terms in genes down-regulated or up-regulated in 3rd trimester EVTs in comparison with 1st trimester EVTs. **B:** Top enriched GO terms in genes down-regulated or up-regulated in 3rd trimester SCTs in comparison with 1st trimester SCTs. In both A and B, VCTs were used to normalize gene expression in each trimester. The top 1,000 genes with the most significant gene expression changes were selected for GO analysis.

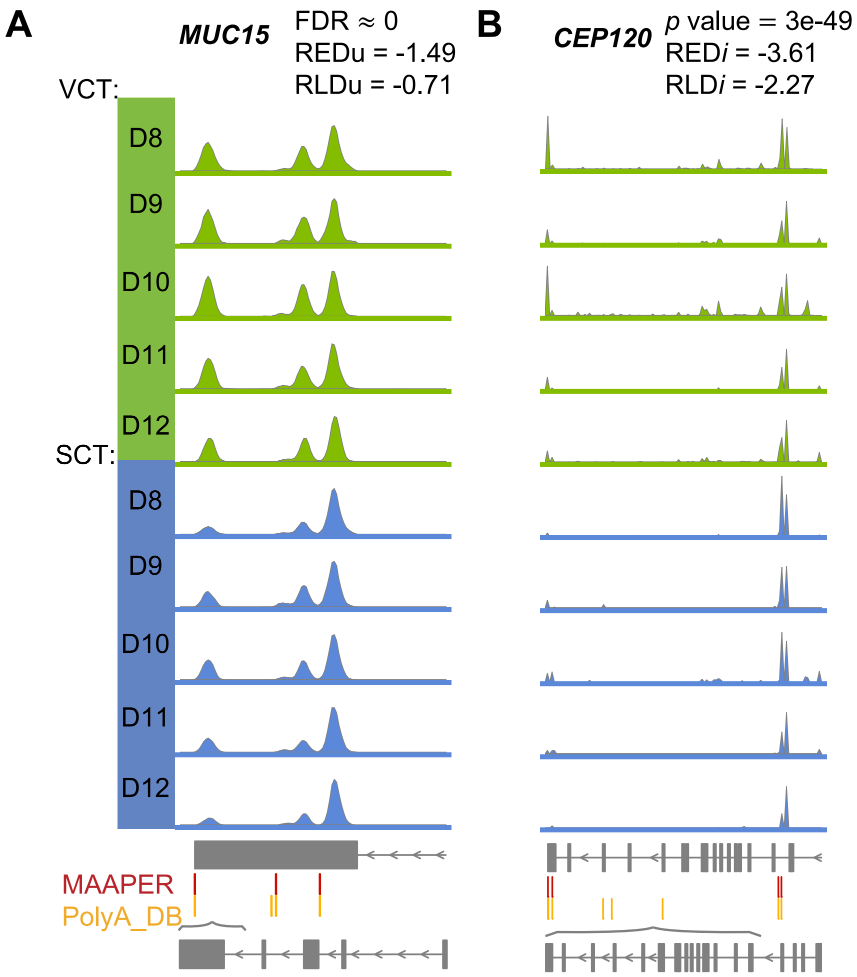

Figure S8. **A-B:** Read alignment tracks of the *MUC15* and *CEP120* genes in VCT and SCT cells.

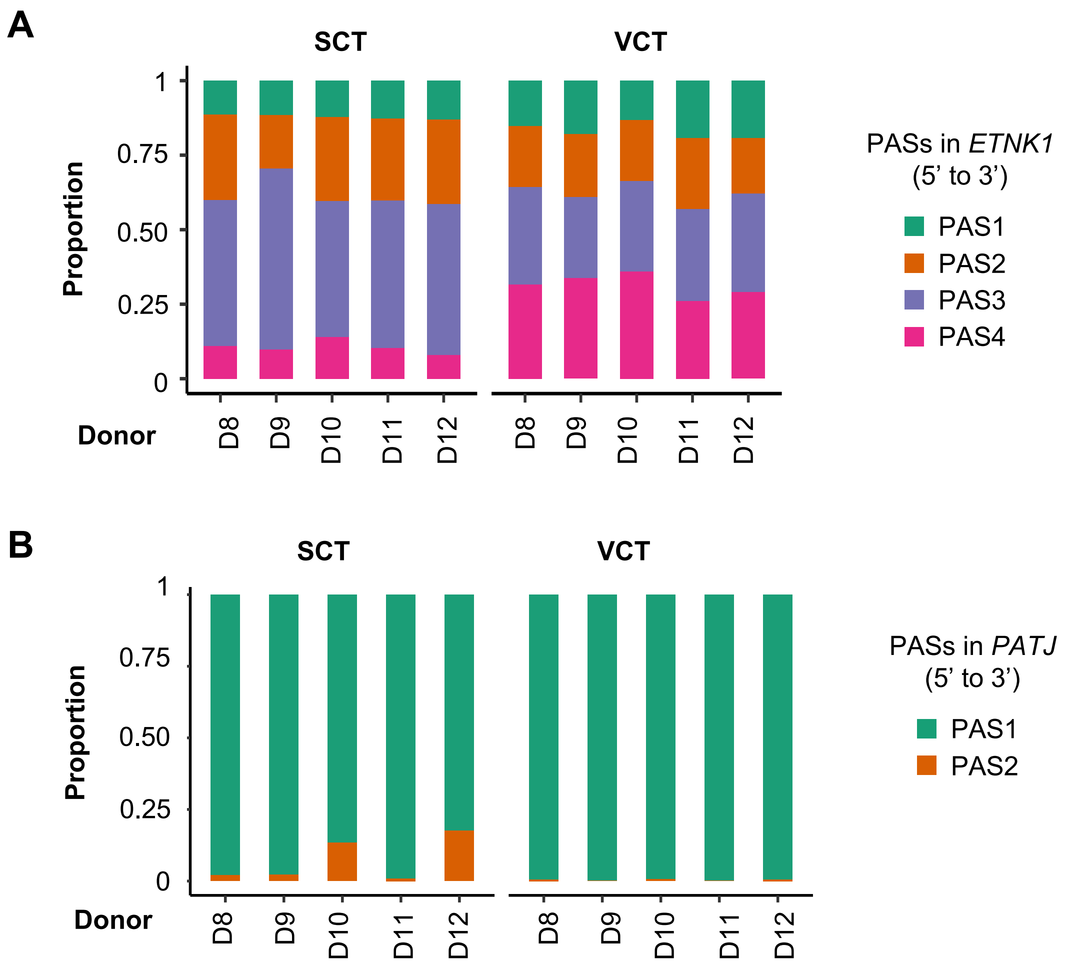

Figure S9. **A:** PAS proportions of the ETNK1 gene in SCTs and VCTs (adjusted *P* = 0.05 in paired test and $7.6{\times}10^{-56}$ in unpaired test). **B:** PAS proportions of the PATJ gene in SCTs and VCTs (adjusted *P* = 0.44 in paired test and $2.2{\times}10^{-308}$ in unpaired test).

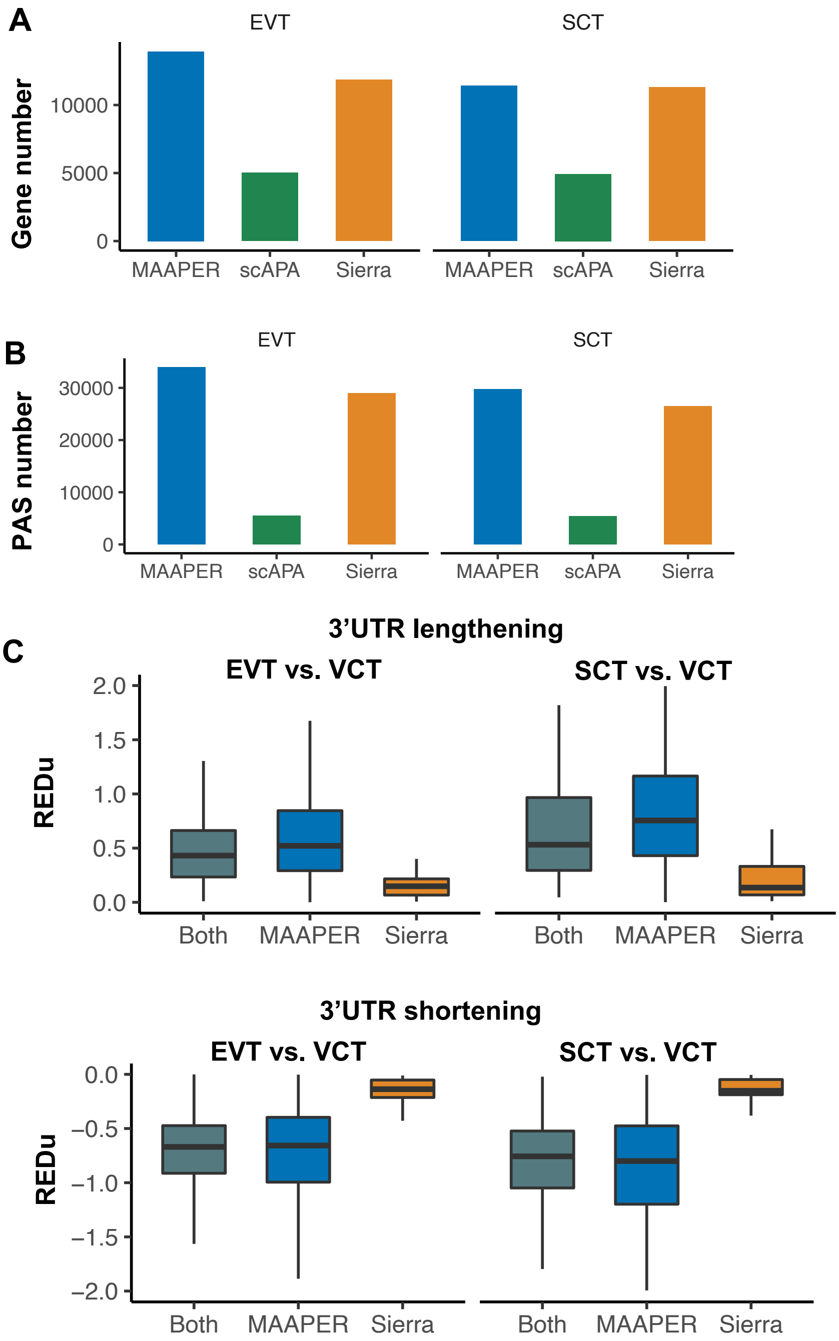

Figure S10. Comparison of PAS prediction by MAAPER, scAPA, and Sierra. **A**: Number of genes detected by the three methods. **B**: Number of PASs detected by the three methods. **C**: REDu scores of genes identified by both MAPPER and Sierra, only by MAAPER, or only by Sierra.

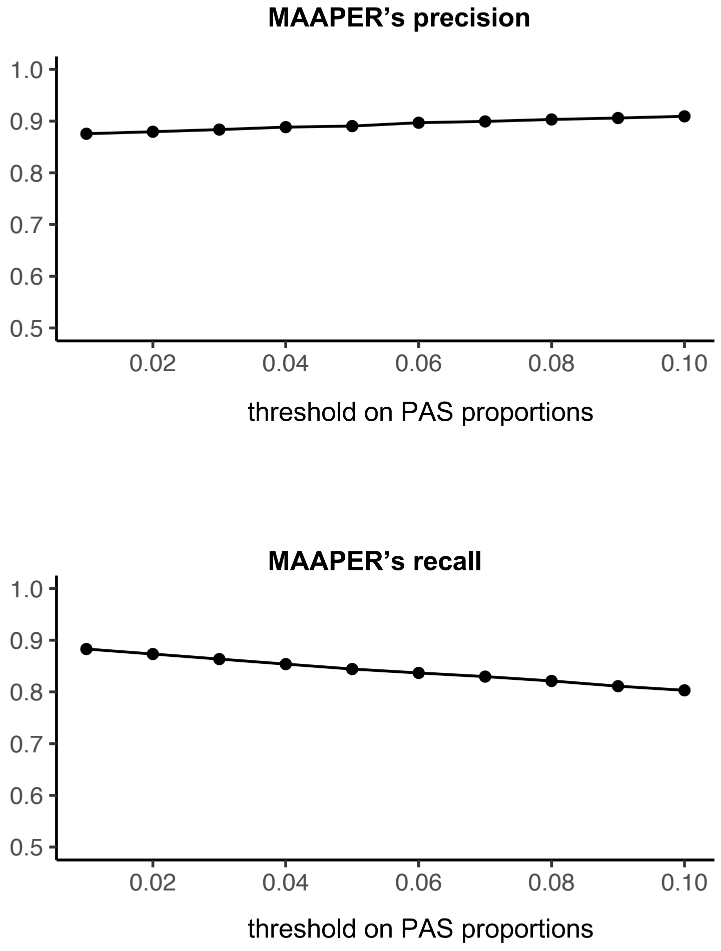

Figure S11. Sensitivity analysis of MAAPER. Precision and recall rates were calculated by comparing MAAPER's results with the identified PASs (restricted to those overlapping with PolyA_DB) from QuantSeq REV data. MAAPER's threshold on PAS proportions varied between 1% and 10%. Analysis was performed using the RC4 sample as an example.
