# Supplementary material for: MAAPER: model-based analysis of alternative polyadenylation using 3′ end-linked reads
Source: Genome Biol. 2021 Aug 10;22:222. doi: 10.1186/s13059-021-02429-5 (PMC8356463; doi:10.1186/s13059-021-02429-5)
Supplement: Supplementary file 2 — Additional file 2. Review history. [file 13059_2021_2429_MOESM2_ESM.docx]

Review History

**First round of review**

**Reviewer 1**

**Were you able to assess all statistics in the manuscript, including the appropriateness of statistical tests used?**

No

**Were you able to directly test the methods?**

No

**Comments to author:**

Li and Tian present a new and general model-based analysis method for alternative polyadenylate (APA) analysis using 3' end-linked reads (MAAPER). The probabilistic model enables MAAPER to accurately predict PASs from nearSite reads as well as identify differential APA event usages. With more and more RNA-seq and single-cell RNA-seq are being generated, this method is helpful to investigate APA across different applications. Overall, this is a very exciting work from a well-known APA laboratory.

Major comments

1. There are multiple methods published to analyze APA from scRNA-seq. Can authors compare MAAPER with other others like Elkon lab (Shulman &Elkon, NAR 2019).

2. Related to question 1, can authors conduct benchmark/simulation to demonstrate the accuracy and sensitivity of MAAPER as well as other methods?

3. What is the analysis speed? How do the computing time and memory requirements scale with increasing number of samples/reads?

**Reviewer 2**

**Were you able to assess all statistics in the manuscript, including the appropriateness of statistical tests used?**

Yes: yes, i was able to assess the statistics.

**Were you able to directly test the methods?**

Yes

**Comments to author:**

This manuscript reports a new method MAAPER to identify polyadenylation sites (PAS) using 3' end-linked reads. This method is based on a probabilistic model relating read density to PAS predictions. It can handle different types of APA events (intronic or exotic), and cases where multiple APA sites exist. The authors showed that MAAPER is applicable to both bulk 3' nearSite reads and single cell RNA-seq data. They also devised statistical tests for differential APA in unpaired or paired experimental designs. The utility of MAAPER to identify PASs in cell populations of scRNA-seq data is a strong feature, and would be useful to others in the field. I have the following comments to improve this work.

MAAPER was designed to analyze nearSite reads generated by specific protocols that produce reads that are close to but do not necessarily contain the PAS. In contrast, there are also protocols that capture onSite reads enabling direct identification of PAS. In bulk RNA sequencing, nearSite protocols seem to be very specialized, and not widely used any more. Thus, MAAPER may not see wide application in this aspect.

As many scRNA-seq data use 3' end counting protocols, MAAPER could be a very useful tool in analyzing scRNA-seq data. However, the authors did not evaluate MAAPER's performance when applied to scRNA-seq data. Since this could be a major application of MAAPER, it's necessary to carry out such evaluations.

In applying MAAPER to scRNA-seq data, it appears that the authors focused on populations of cells by aggregating reads from cells of the same cell type. Could MAAPER be applied to individual cells? If yes, the authors should compare their methods to other methods, such as Sierra and scAPA. If no, the authors should elaborate on this limitation in the manuscript.

A major limitation of MAPPER is that it relies on annotations of known poly A sites. In this manuscript, polyA_DB was used to provide a catalog of known PASs. Since polyA_DB was constructed using bulk data primarily, how applicable is it to PAS in scRNA-seq data that affords opportunities to examine specialized cell types? Does it miss novel PASs that may exist in specific cell types?

The plots in Figure 3B and 4E are very confusing. The median values are quite small (for example, 0.03 or -0.04 in Fig. 4E), whereas the authors claim there exist significant shortening or lengthening of the 3' UTRs or pre-mRNAs. Rather than box plots, it may be better visualizing the positive or negative changes separately, as only those with significant differential changes are shown. It would be good if the authors provide more concrete justifications of their conclusions here.

**Authors Response**

**Point-by-point responses to the reviewers’ comments:**

Reviewer #1:

Li and Tian present a new and general model-based analysis method for alternative polyadenylate (APA) analysis using 3' end-linked reads (MAAPER). The probabilistic model enables MAAPER to accurately predict PASs from nearSite reads as well as identify differential APA event usages. With more and more RNA-seq and single-cell RNA-seq are being generated, this method is helpful to investigate APA across different applications. Overall, this is a very exciting work from a well-known APA laboratory.

*We thank the reviewer for the great enthusiasm about our work!*

Major comments

1.	There are multiple methods published to analyze APA from scRNA-seq. Can authors compare MAAPER with other others like Elkon lab (Shulman &Elkon, NAR 2019).

2.	Related to question 1, can authors conduct benchmark/simulation to demonstrate the accuracy and sensitivity of MAAPER as well as other methods?

*Response:*

*We thank the reviewer for the excellent suggestion! We have carried out thorough comparisons of MAAPER with scAPA (Shulman and Elkon, 2019) and Sierra (Patrick et al., 2020).*

*First, we provide a table summarizing distinct functionalities of the three methods (see Table R1 below and Table 1). Several aspects are of note: 1) only MAAPER can be used on both bulk and single-cell RNA-seq data; 2) MAAPER provides locations of PASs at single-nucleotide resolution, while scAPA and Sierra provide genomic ranges of predicted peaks; 3) While all three methods perform differential analysis using single cell clusters, only MAAPER is capable of paired test, which we believe is very important in patient-based data to account for individual background differences (due to genetics, environmental factors, etc.).*

*Second, to quantitively compare the accuracy of PAS prediction and APA analysis, we applied the three methods to the first-trimester placental samples to compare SCTs or EVTs with VCTs. At the prediction stage, MAAPER on average identified the highest number of genes (12,674) and PASs (31,850). Sierra had a comparable performance (11,591 genes and 28,093 PASs), while scAPA had a much lower detection rate (4,979 genes and 5,479 PASs) (see Figure R1A-C below). For each method, we also calculated the proportion of reads assigned to the predicted PASs using only the detected genes (Figure R1E). On average, MAAPER’s predicted PASs explained 84% of the reads, while scAPA and Sierra explained 65.3% and 57.7%, respectively.*

*Third, we summarized the nucleotide profiles around the predicted PASs (by MAAPER) or the 3’ ends of predicted peaks (by scAPA or Sierra). We found that MAAPER’s predictions were characterized by upstream A-rich and downstream U-rich peaks that are characteristic of genuine PASs. By contrast, the predicted PASs by Sierra or scAPA had less distinctive signals (Figure R1D). In fact, the nucleotide profiles of the PASs predicted by scAPA are a bit troublesome, as A-rich or U-rich peaks, hallmarks of PASs, are not discrete.*

*Figure R1. A: Number of genes detected by the three methods. B: Number of PASs detected by the three methods. C: Number of PASs detected in individual genes by the three methods. D: Nucleotide frequency around the predicted PASs by the three methods. E: Proportion of nearSite reads assigned to the predicted PASs by the three methods.*

*Fourth, we compared significant APA events identified by the three methods. With a universal threshold of FDR-adjusted P = 0.01, scAPA identified only <50 significant APA events in both EVTs and SCTs whereas MAAPER and Sierra on average reported 5,589 and 1,214 significant APA events, respectively. This result indicates that MAAPER is the most sensitive method for APA analysis among the three methods. We found that, in both SCTs and EVTs, >87% of genes with significant 3’UTR changes identified by Sierra were also detected by MAAPER (Figure R2A). Interestingly, the events identified by both methods or by MAAPER only showed similar REDu scores, while events identified by Sierra only had smaller absolute REDu values (Figure R2B), indicating that MAAPER is more selective than Sierra.*

*Figure R2. A: Venn diagrams showing the overlap between significant genes identified by MAAPER and Sierra. B: REDu scores of significant genes identified by both MAAPER and Sierra, or only MAAPER or Sierra.*

*In summary, the comparison results demonstrate higher accuracy and unique applicability of MAAPER in APA analysis as compared with existing methods. We have added the above analysis and results to our revised manuscript (Section “MAAPER improves accuracy of APA analysis with single-cell data”, pages 9-10).*

3.	What is the analysis speed? How do the computing time and memory requirements scale with increasing number of samples/reads?

*Response:*

*We thank the reviewer for raising this important question and have added a comparison between MAPPER, scAPA, and Sierra based on computational efficiency. Our comparison was based on the Ubuntu 16.04.5 system and Intel Xeon "Gold" 6230R processor (16 cores). MAAPER had the lowest memory usage among the three methods and ranked the second in analysis speed (see Table R2 below or Supplementary Table S2).*

*As expected, the computing time of MAAPER increases approximately linearly with increasing numbers of reads (Table R2). The memory usage of MAAPER is less dependent on data size, because it increased from ~30G to ~47G when read number increased from ~56 million to ~1.2 billion. We have added the above analysis and results to our revised manuscript (page 10). We hope these would be useful to MAAPER’s users.*

Reviewer #2:

This manuscript reports a new method MAAPER to identify polyadenylation sites (PAS) using 3' end-linked reads. This method is based on a probabilistic model relating read density to PAS predictions. It can handle different types of APA events (intronic or exotic), and cases where multiple APA sites exist. The authors showed that MAAPER is applicable to both bulk 3' nearSite reads and single cell RNA-seq data. They also devised statistical tests for differential APA in unpaired or paired experimental designs. The utility of MAAPER to identify PASs in cell populations of scRNA-seq data is a strong feature, and would be useful to others in the field. I have the following comments to improve this work.

*We thank this reviewer for the positive comment on the utility of MAAPER.*

MAAPER was designed to analyze nearSite reads generated by specific protocols that produce reads that are close to but do not necessarily contain the PAS. In contrast, there are also protocols that capture onSite reads enabling direct identification of PAS. In bulk RNA sequencing, nearSite protocols seem to be very specialized, and not widely used any more. Thus, MAAPER may not see wide application in this aspect.

*Response:*

*We understand the concern raised by the reviewer. However, based on our analysis of public data repositories, data generated by onSite read-based methods represent a small minority, as compared to those by nearSite methods. While the QuantSeq FWD is not as commonly used as regular full length RNA-seq, the volume of data from scRNA-seq, most of which are nearSite reads, are increasing rapidly. Therefore, we think MAAPER will be widely used, mostly in the scRNA-seq field. We have made this point more clear in our Discussion on page 10.*

As many scRNA-seq data use 3' end counting protocols, MAAPER could be a very useful tool in analyzing scRNA-seq data. However, the authors did not evaluate MAAPER's performance when applied to scRNA-seq data. Since this could be a major application of MAAPER, it's necessary to carry out such evaluations.

*Response:*

*We thank the reviewer for raising this important point. We have addressed this by comparing our method to two other existing tools, scAPA and Sierra. Please see our response to Reviewer 1’s first comment.*

In applying MAAPER to scRNA-seq data, it appears that the authors focused on populations of cells by aggregating reads from cells of the same cell type. Could MAAPER be applied to individual cells? If yes, the authors should compare their methods to other methods, such as Sierra and scAPA. If no, the authors should elaborate on this limitation in the manuscript.

*Response:*

*We appreciate this point raised by this reviewer. We have provided a table summarizing similarities and differences between MAAPER and two other existing methods (see Table R1 above or Table 1). We believe single cell-based APA analysis could have high noise levels based on current scRNA-seq data. We therefore opt for cell cluster-based analysis. We have added the following discussion on page 10 of the revised manuscript:*

*For single-cell analysis, the current MAAPER version uses aggregated single cells of the same type and treats them as pseudo-bulk samples. This approach is in contrast to the two other methods we have compared in this study, namely, scAPA and Sierra. Our rationale is that read counts summarized from scRNA-seq data often contain missing values due to biological and technical reasons [28]. As such, single cell-based APA analysis could have high noise levels [29]. With improvement in sensitivity of scRNA-seq methods, one future direction for MAAPER is to incorporate statistical tests for APA changes in individual cells.*

A major limitation of MAPPER is that it relies on annotations of known poly A sites. In this manuscript, polyA_DB was used to provide a catalog of known PASs. Since polyA_DB was constructed using bulk data primarily, how applicable is it to PAS in scRNA-seq data that affords opportunities to examine specialized cell types? Does it miss novel PASs that may exist in specific cell types?

*Response:*

*We understand this concern. However, using polyA_DB improves data quality and PAS mapping precision, both of which are now made more explicit when we compared MAAPER with Sierra and scAPA. In particular, MAAPER’s predicted PASs on average explained 84% of the reads in the placental data, while scAPA and Sierra explained 65.3% and 57.7%, respectively. The current version of PolyA_DB has a good coverage of PASs, and provides conservation information, which is valuable in analysis. As PAS coverage further improves in PolyA_DB, we expect the sensitivity of MAAPER will also increase. It is also notable that MAAPER is the only method that utilizes quality annotated data for analysis. All other methods, including Sierra and scAPA, are based on de novo PAS prediction. Therefore, in a sense, the unique methodology of MAAPER in using polyA_DB as reference offers users a distinct approach in their work, potentially increasing the robustness of their analysis. We have now made all these points more clear in Discussion (page 10).*

The plots in Figure 3B and 4E are very confusing. The median values are quite small (for example, 0.03 or -0.04 in Fig. 4E), whereas the authors claim there exist significant shortening or lengthening of the 3' UTRs or pre-mRNAs. Rather than box plots, it may be better visualizing the positive or negative changes separately, as only those with significant differential changes are shown. It would be good if the authors provide more concrete justifications of their conclusions here.

*Response:*

*Sorry for the confusion and thanks for the suggestion. In revised Figures 3C, 3E, 4E, and 4G, we now use density plots to show the full data distribution of RED or RLD scores, with median values marked on the side. In Figure 5C, we have also included error bars based on multiple individuals and P values to indicate statistical significance.*

*Please also note that we now split the original RED scores into REDu and REDi in the revised manuscript to make MAAPER analysis more specific for distinct types of APA. REDu measures the relative expression levels between the top two most differentially expressed isoforms in the 3’-most exon only. By contrast, REDi measures the relative expression levels between the top differentially expressed isoform in the 3’-most exon and the top differentially expressed isoform in an intron or middle exon. In the analysis of first trimester placental samples, both SCTs and EVTs showed obvious 3’UTR shortening (Figure 5C), with REDu scores statistically significantly below zero.*

**Second round of review**

**Reviewer 1**

The authors compared MAAPER with the other two methods (scAPA and Sierra) in real data. Though I feel strongly that a simulation is critical for the current APA analysis methods development, this simulation itself can be an independent study alone, thus beyond the scope of this paper.

**Reviewer 2**

I thank the authors for addressing my concerns thoroughly.
